# Synthesis of Naphthoquinone Derivatives: Menaquinones, Lipoquinones and Other Vitamin K Derivatives

**DOI:** 10.3390/molecules25194477

**Published:** 2020-09-29

**Authors:** Margaret Braasch-Turi, Debbie C. Crans

**Affiliations:** 1 Chemistry Department, Colorado State University, Ft. Collins, CO 80525, USA; Maggi.Braasch-Turi@colostate.edu; 2 Cell & Molecular Biology Program, Colorado State University, Ft. Collins, CO 80525, USA

**Keywords:** menaquinone, lipoquinone, synthesis, Friedel-Crafts alkylation, nucleophilic substitution, metal-mediated, electrophilic, pericyclic, and homologation

## Abstract

Menaquinones are a class of isoprenoid molecules that have important roles in human biology and bacterial electron transport, and multiple methods have been developed for their synthesis. These compounds consist of a methylnaphthoquinone (MK) unit and an isoprene side chain, such as found in vitamin K_1_ (phylloquinone), K_2_, and other lipoquinones. The most common naturally occurring menaquinones contain multiple isoprene units and are very hydrophobic, rendering it difficult to evaluate the biological activity of these compounds in aqueous assays. One way to overcome this challenge has been the application of truncated MK-derivatives for their moderate solubility in water. The synthesis of such derivatives has been dominated by Friedel-Crafts alkylation with BF_3_∙OEt_2_. This attractive method occurs over two steps from commercially available starting materials, but it generally produces low yields and a mixture of isomers. In this review, we summarize reported syntheses of both truncated and naturally occurring MK-derivatives that encompass five different synthetic strategies: Nucleophilic ring methods, metal-mediated reactions, electrophilic ring methods, pericyclic reactions, and homologation and side chain extensions. The advantages and disadvantages of each method are discussed, identifying methods with a focus on high yields, regioselectivity, and stereochemistry leading to a detailed overview of the reported chemistry available for preparation of these compounds.

## 1. Introduction

Menaquinones are hydrophobic, isoprenoid molecules containing a methylated naphthoquinone unit and an isoprene side chain which constitutes a subgroup of lipoquinones [1,2,3,4,5,6,7]. There are two major structural subgroups of lipoquinones. Ubiquinones (or benzoquinones, U) are generally found in eukaryotes and Gram-negative prokaryotes, and menaquinones (or naphthoquinones, MK) generally found in Gram-positive prokaryotes. In humans, menaquinones have several biological properties, including facilitating blood coagulation. In bacteria, menaquinones are essential molecules that shuttle electrons between the membrane-bound protein complexes acting as electron acceptors and donors in the respiratory electron transport system and consequently resulting in ATP synthesis. Menaquinones are also referred to as vitamin K_2_, a subgroup of the class of compounds categorized as vitamin K. The preparation and studies of some of these compounds have been extensive and include the preparation both by fermentation and synthesis [8,9,10]. The major structural variation in menaquinones involves the isoprene side chain; that is the number of isoprene units and saturation in the side chain [11]. Although these minor structural changes are deceptively simple, the specific stereochemistry of each is required to maintain their biological action. Hence, the synthesis of these compounds requires attention to detail and often meticulous purification, which consequently leads to a decrease in yields of the desired compounds. In the following review, we will summarize reported methods and discuss the advantages and disadvantages of each, as well as identify the most suitable methods within each synthetic strategy.

**Figure 1:**
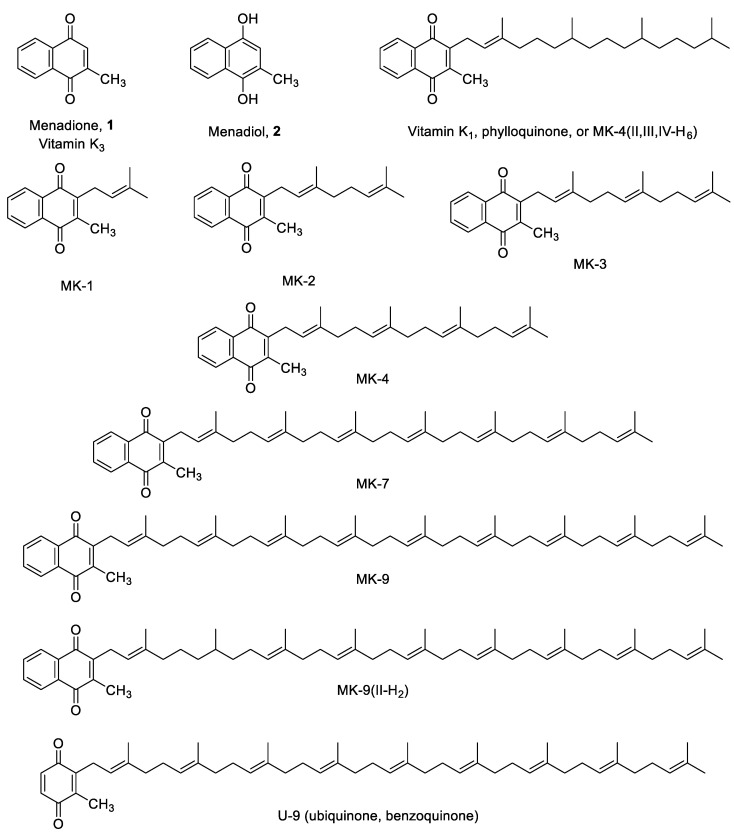
Common menaquinones and ubiquinones.

Vitamin K_2_ and vitamin K_1_ (also referred to as phylloquinone) are natural vitamins and together make up the family of compounds known as vitamins K. The variety of the structures of vitamin K_2_ depend on the number of isoprene units in the side chain. In Figure 1, we show the structures of menadione (vitamin K_3_) **1**, menadiol **2**, vitamin K_1_, and menaquinone (MK) derivatives with 1 to 9 isoprene groups in the side chain. The abbreviation used for the menaquinones in this manuscript will indicate the number of isoprene units in the side chain. For example, MK-1 describes a menaquinone with one isoprene unit, and MK-9, the major MK-derivative found in the *Mycobacteria*, contains nine isoprene units [12]. If the MK-derivative contains isoprene units that are saturated, it will be indicated by the addition of a Roman numeral to specify the location of the isoprene unit numbered from the naphthoquinone. For example, MK-9(II-H_2_) is the major MK-derivative active as the electron transport agent in the *Mycobacterium tuberculosis.* It is a MK-9 derivative with the second isoprene group saturated [6]. When more than one isoprene units are saturated, such as the three units in vitamin K_1_, the nomenclature will identify the location of the saturations using Roman numerals and the number of H-atoms added, such as MK-4(II,III,IV-H_6_) indicating the first isoprene unit is still unsaturated [11].

The biological activities of these compounds have been studied in-depth, and most reviews that been reported have a biological-biomedical focus. In general, terpenes and isoprenoid compounds have been widely reviewed for many uses, including pharmaceutical, flavor fragrance, and possible applications in biofuel industries [13]. Naphthoquinones have been assessed for their biological activity against cancer [14], cardiovascular disease [15], tuberculosis [16,17], diabetes [18], kidney function [19], and age-related diseases [20]. The most well-known members of the naphthoquinone family are the compounds known as vitamin K. Much work has been done in this area comparing the health effects of synthetic vitamin K analogs to naturally-derived analogs [21]. The biosynthesis of vitamin K analogs have been mapped out within bacteria [22], particularly with respect to intestinal bacteria in relation to coagulation homeostasis [23], and discovery of possible drug targets to inhibit electron transport systems [24]. Another important effect caused by vitamin K_1_ is the regulation of calcium uptake, particularly in bone of humans and other mammals [25].

In addition, menaquinones are hydrophobic molecules that shuttle electrons between the membrane-bound protein complexes acting as electron acceptors and donors in the respiratory electron transport system facilitating oxidative phosphorylation in bacteria [16,22]. In *Mycobacteria,* a menaquinone headgroup is required, whereas in a bacterium such as *E. Coli*, both menaquinones and ubiquinones (U-9, Figure 1) are able to serve this function [6]. In the case of *Mycobacterium smegmatis,* MK-9(II-H_2_) was proposed as a contextual virulent factor [6], but this suggestion was later rejected when the presence of inactive MenJ protein was shown to prevent infection by the bacterium. Thus, the protein rather than the substrate was found to be the contextual virulent factor [26]. Development of inhibitors for the biosynthesis of menaquinones or other isoprene-derivatives has been explored as potential treatments [27,28].

### 1.1. Properties and Biological Function of Menaquinones

**Figure 2:**
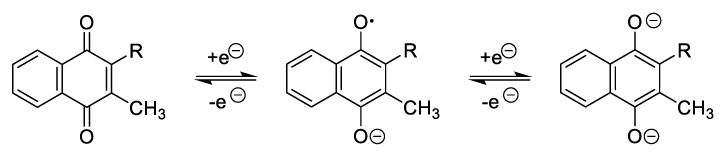
The redox reaction of menaquinones showing the two steps observed in aprotic organic solvent; in the presence of a protic solvent it occurs in only one step, but the overall reaction is conserved.

The structural and redox properties of menaquinones are key for their biological function. The quinone group of the menaquinones is reduced through a two-electron transport system to form a radical anion after the addition of the first electron [29,30,31]. After addition of the second electron, a dianion catecholate is formed, which then forms a catechol in the presence of a protic solvent (Figure 2). The two intermediates are sufficiently long-lived to be observed in aprotic solvents because the proton transfer step is slow. However, in a protic solvent the proton transfer is faster, and protonation of the intermediate radical ion occurs quickly, and the second intermediate is not long-lived enough to be observed using electrochemistry. For MK-derivatives in cellular systems, the presence of H_2_O renders the redox chemistry a one step process. Even though menaquinones are located in the membrane, H_2_O is an accessible proton source. It is highly likely that the redox reaction involving fewer intermediates is important for the biological properties of these compounds. Our group has been investigating the redox properties of some menaquinone systems and found distinctly different properties in organic solvents where the MK-derivatives are soluble [12], compared to aqueous systems where the menaquinone will be interacting with a membrane or a model membrane system, such as a liposome [32].

The location of menaquinones in biological systems is associated with the membrane due to their hydrophobicity. These compounds have been presumed to reside inside the membrane; however, few studies have been done to investigate the specific interaction. Some computational studies have examined the association with the membrane because it is critical to understand the interaction for the electron transport action of menaquinones in bacterial systems [33]; however, less work has been done experimentally. A range of studies have been performed with ubiquinone derivatives, and evidence for the membrane association has been reported both in computational and experimental systems [34,35,36]. On the other hand, significantly less work has been done with the menaquinone system, but so far, the reports support the presumptions often that the menaquinone is associated with the membrane.

Despite the polarity of the quinone group, these molecules are very hydrophobic, even though this property does vary with the length and the nature of the isoprene side group. We have recently found by synthesis of truncated MK-derivatives that only MK-1, MK-2, and MK-3, including derivatives with fully or partially saturated counterparts, are soluble in aqueous solution [12,37,38]. This means that assays with MK-4, even though it is known as an enzyme substrate *in vivo*, may not demonstrate enzyme activity even if the aqueous assay includes surfactants [39]. This experiment was attempted for MenJ, the membrane bound enzyme reported to stereospecifically hydrogenate the second isoprene unit of MK-9 in *Mycobacterium tuberculosis* and *Mycobacterium smegmatis*. The inability of the isolated enzyme to saturate MK-4 was attributed to the poor solubility of MK-4 in vitro since it was following the study that demonstrated in vivo activity. These experiments document the need for use of the truncated MK-derivatives and caution the use of computational methods for evaluation of these systems. For example, the anticancer properties of MK-1 through MK-7 were investigated with a series of five cancer cell lines [40]. MK-1, MK-2, and MK-3 were shown to be cytotoxic, however, MK-4, MK-5, MK-6, and MK-7 did not exhibit any cytotoxic activity against any of the cell five cell lines. Based on the studies with MenJ, it seems likely that the anticancer effects of longer MK-derivatives are a consequence of the physical properties of these derivatives, and any correlations and conclusions regarding the measured activities are not based on the true cytotoxicity of these compounds, but a reflection of their hydrophobicity.

Due to the distinct hydrophobicity of menaquinones, assaying these substrates in vitro is often critical and or convenient in biological studies. Since assays are often done in aqueous solution, this can cause problems if the substrates are menaquinones with longer isoprene side chains, because the longer naturally occurring MK-derivatives are often insoluble in aqueous solution. Contrary to common belief, the addition of surfactants in such assays is often insufficient to solubilize these compounds. The approach we have used to overcome this challenge is to synthesize water soluble, truncated MK-substrates [41]. To this end, the small, truncated MK-derivatives, MK-1 through MK-3, are somewhat soluble in aqueous assays with added surfactants, which makes these MK-derivatives excellent substrates for biological studies. We have recently used this approach in studies of MenJ documenting the effectiveness of this approach [26].

### 1.2. Synthetic Strategy for the Preparation of Menaquinones

**Scheme 1:**
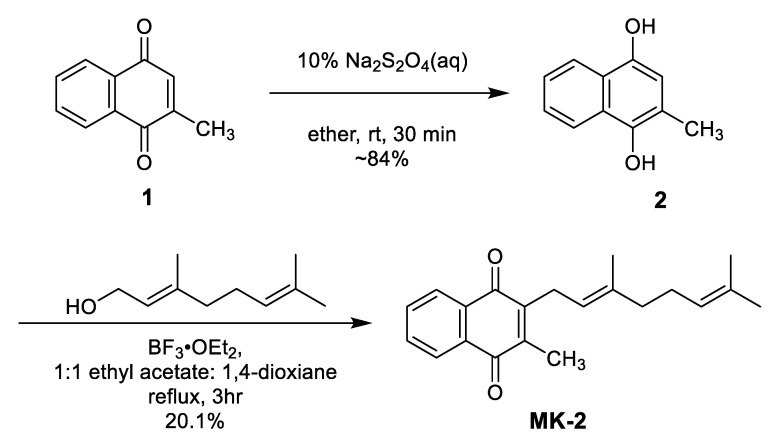
Friedel-Crafts alkylation using BF_3_∙OEt_2_ and commercially available materials [12].

Contemplating the many synthetic strategies available, consideration of the structure and the application of the MK-derivatives may be important when choosing the appropriate method for synthesis. So far, our group has focused on the preparation of truncated MK-derivatives, where the syntheses are relatively short and direct [12,37,38]. Therein, we used Friedel-Crafts alkylation using BF_3_∙OEt_2_ as a Lewis acid catalyst. This use of electrophilic aromatic substitution is one of the most common across the literature for the synthesis of this family of compounds. This is most likely because it occurs over two steps from commercially available starting materials. For example, for our synthesis of MK-2, we begin by reducing menadione **1** with 10% Na_2_S_2_O_4_ at room temperature for 30 min to produce menadiol **2** in ~84% yield (Scheme 1) [12]. Then menadiol **2** underwent alkylation with commercially available geraniol in the presence of BF_3_∙OEt_2_, producing MK-2 in 20.1% yield. This approach may be direct, but it generally results in low yields (< 30%) and a mixture of isomers. Although biological studies do not require very much sample, consistently low yielding reactions are not suitable for multi-step syntheses at large scales, such as longer MK-derivatives where the key step is a Friedel-Crafts alkylation.

**Figure 3:**
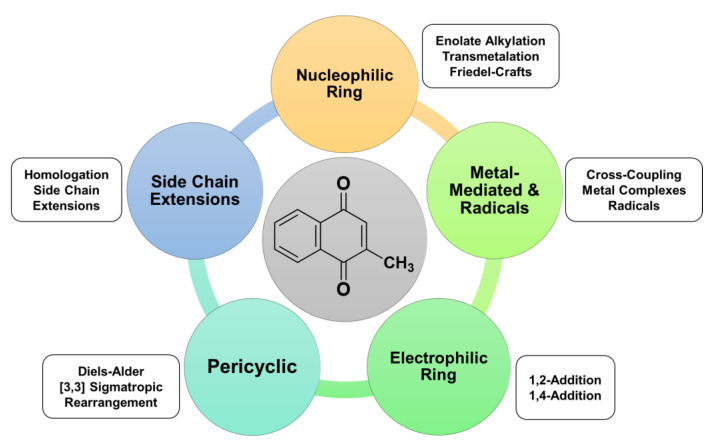
5 Main synthetic strategies from across the literature for the synthesis of menaquinone derivatives.

Two reviews [42,43] have been published that focus on the synthesis of the biologically relevant vitamin K_1_. In this review, we summarize the syntheses of MK-derivatives reported in recent history. This includes menaquinones of varying side chain lengths, which include many types of vitamin K_2_ derivatives, as well as vitamin K_1_. This review will highlight the five most common synthetic strategies used for preparation of truncated and full-length MK-derivatives: nucleophilic ring methods, metal-mediated and radical reactions, electrophilic ring methods, pericyclic reactions, and side chain extensions (Figure 3). Each class of reactions will be summarized, identifying the advantages and disadvantages of each. This will allow an evaluation of each strategy based on the overall yields of the synthesis, regioselectivity, and the stereoretention of the first isoprene (α) unit from the ring. Other comparisons concerning the number of steps, competing side reactions, and safety will also be presented, which will result in a summary identifying the most attractive synthetic strategies from each category for preparation of this class of compounds.

## 2. Nucleophilic Ring Methods

### 2.1. Enolate Alkylation

**Scheme 2:**
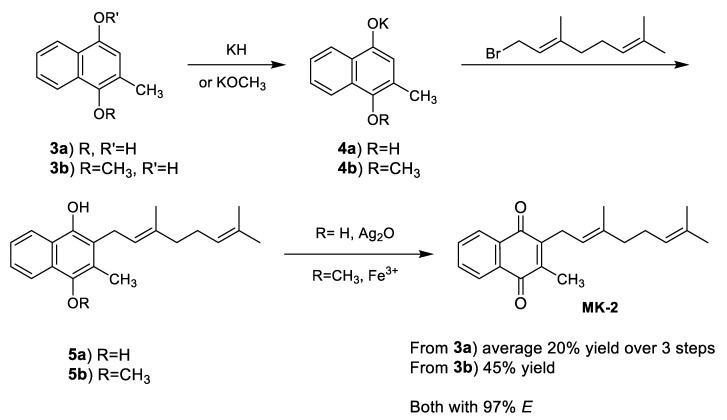
Synthesis of MK-2 via enolate alkylation [44].

In 1974, Snyder and Rapoport published a comprehensive report detailing their attempts to synthesize menaquinones [44]. The main goal of their syntheses was to retain the stereochemistry of α-isoprene double bond. The first approach used enolate chemistry to alkylate the C3 position of menadiol **2**. Beginning with menadiol **3a**, the potassium salt **4a** was formed using potassium hydride or potassium methoxide (Scheme 2). It was suspected that Claisen alkylation had occurred, but upon further analysis it was concluded that the reaction proceeded via enolate alkylation to form **5a** with 97:3 *E/Z*. Oxidation of the ring in the presence of Ag_2_O formed MK-2 in an average of 20% yield over three steps. The authors postulated the lower yield was due to competing Friedel-Crafts alkylation occurring on the C2 position. To prevent competition, the authors redeveloped the route using C1 methyl ether protected menadiol **3b**. Conversion of **3b** to MK-2 yielded 45% with 97% *E* alkene (Scheme 2). Despite the improved yield, the synthesis of **3b** was more complicated than the authors originally thought. Therefore, this route was abandoned for one with more accessible starting materials.

**Scheme 3:**
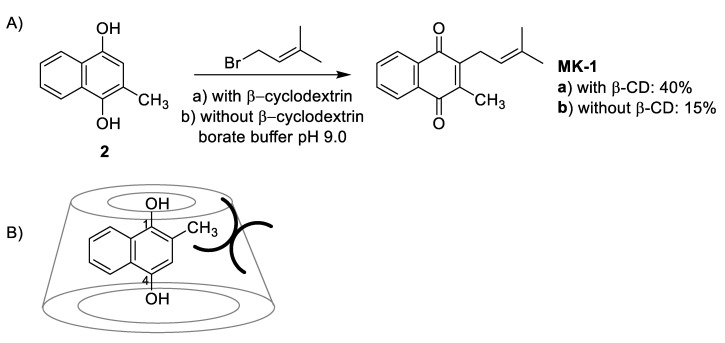
(**A**) Synthesis of MK-1 using β-cyclodextrin (β-CD) as an inclusion catalyst. (**B**) Steric hinderance (shown in bold) of the β-cyclodextrin scaffold preventing C2 alkylation [45,46].

A few years later, Tabushi et al. published on the allylation of naphthoquinones using β-cyclodextrin as an inclusion catalyst, especially for synthesis of vitamin K_1_ [45,46]. Menadiol **2** was alkylated at the C3 position in mild basic conditions, borate buffer (pH 9), with and without β-cyclodextrin (Scheme 3A). The yields of MK-1 were found to be 40% and 15%, respectively, showing a significant decrease in yield without β-cyclodextrin. The only byproduct observed was menadione **1** in 49% and 28% yield, respectively.

The results strongly indicate that β-cyclodextrin played significant role catalyzing this reaction. The authors noted the selectivity of the alkylation behaved as if it were a ligase and or oxidase. Due to the semi-conical structure of β-cyclodextrin, menadiol is surrounded by the hydrophobic cavity. The C1 and C4 hydroxy groups interact with the hydrophilic exterior (Scheme 3B). The C1 hydroxy group hypothesized to be hydrogen bonding on the narrower end of the scaffold. The hydrogen bonding interactions were found to lower the pKa of the opposite hydroxy group at the C4 position. Unbound menadiol has a pKa of 9.45, and bound menadiol was calculated to have a pKa of ~8.90 [46]. The decrease in pKa was said to enhance the nucleophilicity of the partially charged carbanion on the C3 position. Deprotonation of the C4 hydroxy group in pH 9 medium created a more nucleophilic enolate, alkylating at the C3 position. The authors did not observe any C2-alkylated product in their trials. They postulated the sterically hindered, semi-conical shape of the β-cyclodextrin scaffold prevented C2 alkylation (Scheme 3B). Menadione **1** was the only other product formed. In both trials, **1** was produced in higher yields than MK-1. In protic solvents, menadiol **2** will spontaneously oxidize to form menadione **1**. In the presence of an enzyme-like cavity of β-cyclodextrin, the transformation was most likely accelerated in the buffer solution. Although menadione **1** was formed in higher yields, it can be recovered and recycled for subsequent use.

### 2.2. Transmetalation

**Scheme 4:**
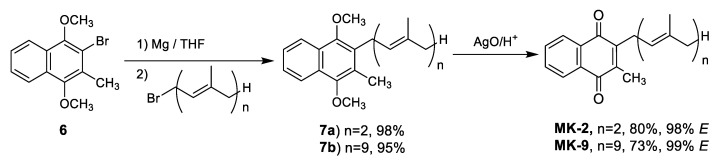
Synthesis of MK-2 and MK-9 using Grignard reagents [44].

After marginal success with enolate alkylations, Snyder and Rapoport shifted their focus towards more direct nucleophilic methods [44]. Bismethyl ether 2-bromomenadiol **6** was transformed into 2-metallo derivatives using lithium, magnesium, and copper to react with a variety of electrophiles (Scheme 4). The authors were originally interested in using aldehydes as electrophiles; however, their attempts were unsuccessful. Removal of the resultant benzyl alcohol led to the formation of a vinyl alkene or isomerization of the α-isoprene double bond. To avoid this, prenyl halide substrates were used instead. Preliminary reactions were performed to assess the stereoretention of the α-isoprene double bond for each 2-metallo derivative. All three 2-metallo derivatives left the α-isoprene double bond virtually unaffected. Organolithium yields were 10% and 65% for geranyl chloride and bromide, respectively, leading to the exclusive use of prenyl bromide electrophiles. Lithium organocuprate and organocuprate yields were 74% and 82%, respectively. The Grignard reagent was formed in 92% yield. Snyder and Rapoport continued their studies using the Grignard reagent.

To examine the utility of this method, the authors synthesized MK-2 and MK-9 (Scheme 4). The Grignard reagent was produced by stirring **6** with Mg turnings in dry THF. Geranyl bromide and solanesyl bromide were added to the solution, resulting in the respective alkylated products, **7a** and **7b**. Removal of the bismethyl ethers and oxidation of the ring were achieved with AgO in acidic conditions yielding 80% and 73% for MK-2 and MK-9, respectively. Both products retained the stereochemistry of the α-isoprene double bond, each with ≥98% *E* alkene.

**Scheme 5:**
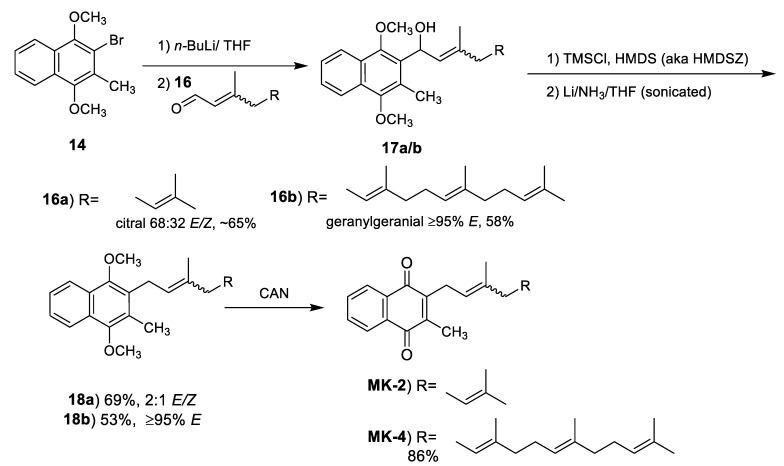
Synthesis of MK-2 and MK-4 using BIHY conditions [47,48,49].

Unlike Snyder and Rapoport, Saá and coworkers were successful using aldehydes as electrophiles in the early 1990s. This challenge was overcome with the use of stereocontrolled Birch hydrogenolysis conditions (BIHY) to selectively remove silyl ether protected *(E)-* or *(Z)-*α-alkenylbenzyl alcohols [47,48,49]. The authors applied this method towards the synthesis of MK-2 and MK-4 (Scheme 5). Using the same starting material as Snyder and Rapoport, bismethyl ether 2-bromomenadiol **6** underwent lithium-bromide exchange to form the organolithium reagent. Commercially available aldehydes, citral **8a** (68:32 *E/Z*) and geranylgeranial **8b** (≥ 95% *E*), were used without further purification to assess the stereoretentive abilities of BIHY conditions. Upon nucleophilic attack, the resultant benzylic alcohols **9a** and **9b** were formed in ~65% and 58% yield, respectively. The alcohols were protected with TMSCl in the presence of HMDS, as used in previous reports [48,49]. In the literature, the authors used the abbreviation “HMDSZ” instead of “HMDS”, which is now more the commonly used abbreviation. The protected alcohol was then reduced in the presence of lithium metal and liquid ammonia to produce the free methylene **10**. With this method, the resulting stereochemistry of **10a** and **10b** α-isoprene double bonds were determined to be 2:1 *E/Z* and ≥95% *E* alkene, respectively. These results reflect the stereochemistry of the aldehyde precursors and demonstrate the remarkable stereoretention of BIHY. The methyl ether protecting groups were removed using CAN to produce MK-2 and MK-4. Only the reported yield was for MK-4 in 86%.

**Scheme 6:**
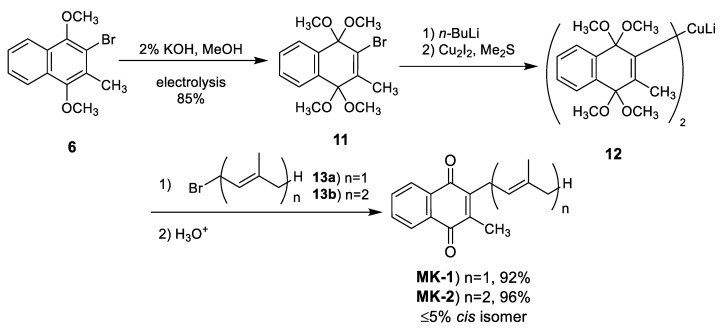
Synthesis of MK-1 and MK-2 featuring electrolysis as a protection method [50,51].

Further improvement upon the strategies established by Snyder and Rapoport was reported by Swenton and coworkers in 1977 [50]. Therein, preliminary results were published using electrolysis to protect bismethyl 2-bromomenadiol **6** as bismethyl ketals. The authors used lithium organocuprate nucleophiles instead of Grignard reagents. In 1980, the authors published a complete study towards the synthesis of menaquinones [51]. Bismethyl ether 2-bromomenadiol **6** underwent electrolysis in a divided cell with 2% KOH and methanol, producing bisketal **11** (Scheme 6). Lithium-bromide exchange in THF and subsequent transmetalation with cuprous iodide produced the desired lithium organocuprate dimer **12**. The corresponding electrophiles, prenyl bromide **13a** and geranyl bromide **13b**, were added to the solution and immediately carried forward to hydrolysis without purification. MK-1 and MK-2 were formed in 92% and 96% yield, respectively. The authors did not observe any evidence of the *Z* isomer in NMR; however, its absence could not be concluded.

### 2.3. Friedel-Crafts Alkylation

Throughout all the strategies described in this report, the Friedel-Crafts alkylation is by far the most popular across the literature. The most common Lewis acid catalyst used for this specific transformation is BF_3_∙OEt_2_, which became popular after Lindlar’s 1953 patent detailing its usage [52]. Two more reports of its use specifically towards the synthesis of vitamin K_1_ were published in the following year. First, Isler and Doebel detailed the synthesis of vitamin K_1_ and various other racemic derivatives [53]. Second, Hirschmann et al. reported the comparison of different Lewis and Brønsted-Lowry acid catalysts towards the synthesis of vitamin K_1_ [54] in an effort to improve upon existing reported methods at the time [55,56,57,58,59,60,61]. Most notably among those methods, Fieser reported the condensation of menadiol and phytol in the presence of oxalic acid with overall yields of 25–30% [55]. Much of the yield was lost to undesired side products, such as phytadiene and the C2-alkylated product [62]. Many of the early reports do not use protecting groups or other functional handles to induce regioselectivity.

**Scheme 7:**
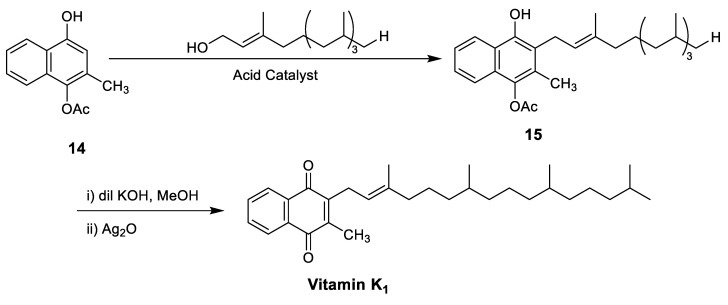
Synthesis of vitamin K_1_ using Friedel-Crafts alkylation with different acid catalysts [54].

To address the regioselectivity challenge, Hirschmann et al. designed monoacetate **14** to influence C3 alkylation (Scheme 7). Monoacetate **14** underwent condensation with phytol in the presence of an acid catalyst to form alkylated product **15**. Potassium acid sulfate, oxalic acid, Duolite C-60 cation exchange resin, and BF_3_∙OEt_2_ produced varied results, as outlined in Table 1. Removal of the acetate protecting group was achieved using Claisen alkali conditions (dilute KOH in methanol), and oxidation with Ag_2_O work up formed vitamin K_1_.

**Scheme 8:**
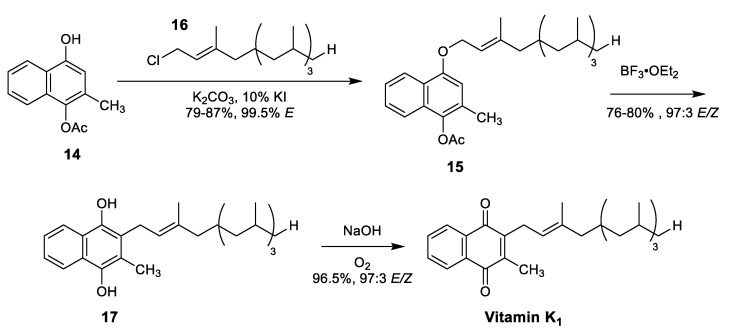
Synthesis of vitamin K_1_ featuring an intramolecular Friedel-Crafts alkylation [63].

In 1990, Schmid et al. published a comprehensive report on the synthesis and analysis of all four stereoisomers of (*E*)-vitamin K_1_ [63]. The synthesis of the naturally occurring stereoisomer showcased a unique transformation using BF_3_∙OEt_2_ as the catalyst (Scheme 8). Starting with monoacetate **14**, the free phenol was alkylated with phytyl chloride **16** and potassium carbonate in 79–87% yield with 99.5% *E* alkene. In the presence of BF_3_∙OEt_2,_ the O-alkylated product **15** was thought to have undergone a Claisen rearrangement to form C-alkylated product **17**, but upon further analysis, the reaction was determined to proceed via and intramolecular Friedel-Crafts mechanism. The C-alkylated product **17** was formed in 76–80% yield and the *E*/*Z* ratio was found to be 97:3. This kind of intramolecular transformation was first reported by Yoshizawa et al. in 1982 on ubiquinone derivatives [64]. Removal of the acetyl group in basic conditions produced vitamin K_1_ in 96.5% yield based on HPLC. The *E*/*Z* ratio of the α-isoprene double bond was determined to be 97:3, unchanged from the previous step. In 2003, the authors of a vitamin K_1_ syntheses review [42] commented that this route has received little attention.

**Scheme 9:**
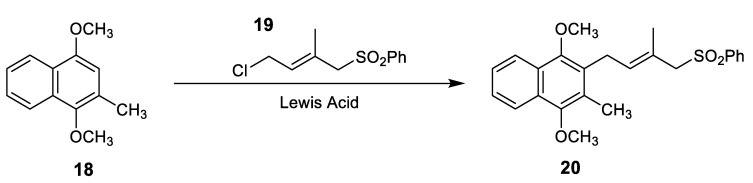
Synthesis of truncated MK-derivatives using Friedel-Crafts alkylation [65].

Allyl alcohols have a reputation for being intrinsically unstable towards alcohol rearrangement. To circumvent this issue, Min et al. used prenyl chlorides instead [65]. Menadiol dimethyl ether **18** was reacted with prenyl chloride **19** in presence of BF_3_∙OEt_2_ to form sulfonyl intermediate **20** (Scheme 9). Truncated prenyl chain **19** allowed for further functionalization of the side chain to make more diverse analogs, which will be discussed in detail later in Section 6.2. The authors assessed the efficacy of a large scope of Lewis acids, which are summarized in Table 2. Based on these results, AlCl_3_ performed the best with 72% yield and all *E* configuration of the α-isoprene double bonds.

**Scheme 10:**
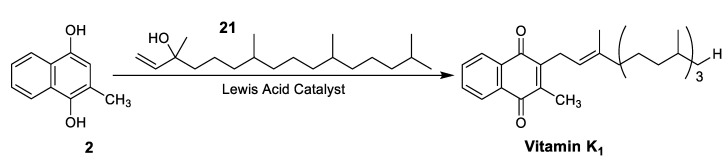
Synthesis of vitamin K_1_ using partly hydroxylated magnesium and aluminum fluorides [66].

Despite the historical popularity of BF_3∙_OEt_2_, the competition between C2/C3 alkylation continued to be a persistent challenge. Various types of protecting groups have been used to prevent competition with varying success. Due to competing side reactions, its use in industrial applications has been accused of being wasteful, inspiring researchers to identify new, more sustainable Lewis acids to minimize byproducts and maximize yield. Coman et al. developed a new class of heterogenous, partly hydroxylated magnesium and aluminum fluorides to address such concerns [66]. The authors predict that this class of catalysts will replace homogenous BF_3∙_OEt_2_ in industrial applications. Vitamin K_1_ was synthesized to illustrate this concept starting with menadiol **2** and isophytol **21** (Scheme 10). The reactions resulted in 100% conversion of the starting material with each catalyst. The yields were low and showed considerable formation of byproducts, mainly chromanol and C2-alkylated product, as shown in Table 3. The catalyst, MgF_2_-57 provided the best results with respect to vitamin K_1_, yielding 26.5%, which is comparable to BF_3_∙OEt_2_.

**Scheme 11:**
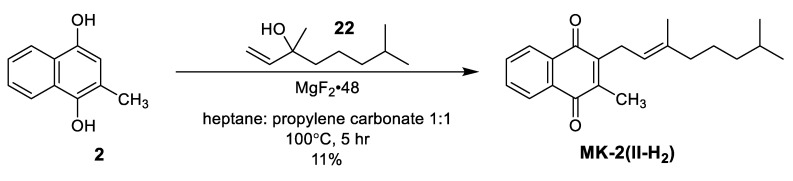
Synthesis of MK-2(II-H_2_) using using MgF_2_-48 as a catalyst [38].

Recently, we synthesized fully and partially saturated MK-derivatives in an effort to understand their structural and electrochemical properties in model membranes [12,37,38]. For the synthesis of MK-2(II-H_2_), the condensation of isophytol **22** and menadiol **2** was accomplished in 11% yield using MgF_2_-48, a Coman et al. inspired catalyst (Scheme 11). Before purification, the crude yield was determined to be 60%, consisting of regio- and stereoisomers. The regioisomers separated readily via column chromatography; however, the *E*/*Z* isomers of the C3-product required thorough purification using preparative TLC. Therefore, these stereoisomers are purified on demand for the biological studies.

### 2.4. Summary

The three main nucleophilic ring methods throughout the literature include enolate alkylation, transmetalation of bromonaphthoquinone derivatives, and Friedel-Crafts alkylation. The advantages and disadvantages of each have been outline in Table 4. Out of all nine methods presented, only three of them reported overall yields greater than 80%. These methods were Snyder and Rapoport’s aryl-Grignard reaction with prenyl bromides, Swenton and coworker’s use of electrolytically protected lithium organocuprate, and Schmid et al.’s unique intramolecular Friedel-Crafts alkylation. The first two methods also demonstrated exemplary regiocontrol due to lithium-bromide exchange with of bismethyl ether 2-bromomenadiol **6**. The other methods produced regioisomers owing to prominent competition between C2 and C3 alkylation. Many methods did not utilize protecting groups or directing group manipulations to influence regiocontrol. For most of these methods, the chosen electrophiles were prenyl halides, which left the α-isoprene double bond virtually undisturbed. However, in the case of Tabushi et al., the authors only synthesized MK-1; therefore, the stereochemical implications of the method were not addressed.

## 3. Metal-Mediated and Radical Reactions

### 3.1. Cross-Coupling

**Scheme 12:**
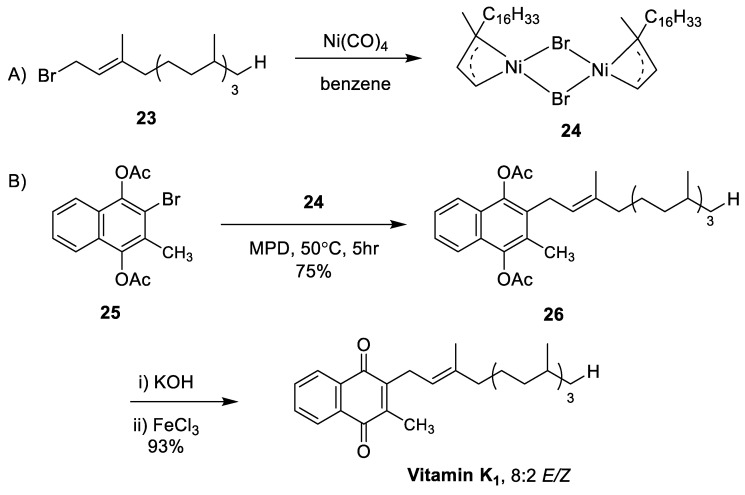
(**A**) Formation of π-allylnickel complex. (**B**) Synthesis of vitamin K_1_ using π-allylnickel cross-coupling [69].

**Scheme 13:**
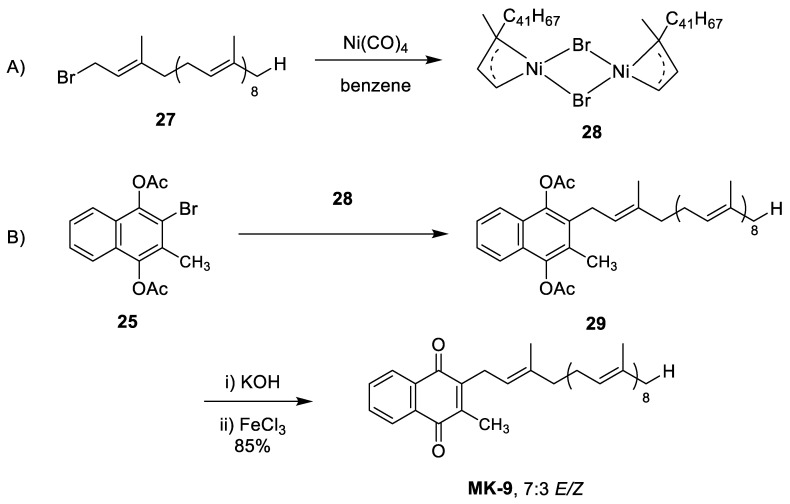
(**A**) Formation of π-allylnickel complex. (**B**) Synthesis of MK-9 using π-allylnickel cross-coupling [69].

After the incorporation of transmetalation, synthetic efforts were shifted to investigate cross-coupling reactions. In 1973, Sato et al. used π-allylnickel chemistry to synthesize vitamin K_1_ and MK-9 after initial success with MK-1 [69]. For this method, π-allylnickel complexes **24** and **28** were formed in situ using allyl bromides **23** and **27** with Ni(CO)_4_ (Scheme 12A and Scheme 13A). Vitamin K_1_ was synthesized from diacetate **25** and π-allyl complex **24** in MPD at 50 °C for 5 h to form the expected alkylated product **26** in 75% yield (Scheme 12B). The acetate protecting groups were removed in mild basic conditions, and the oxidation was achieved using FeCl_3_, producing vitamin K_1_ in 93% over two steps. The observed *E/Z* ratio was 8:2 with respect to the α-isoprene double bond. For MK-9, the same starting material diacetate **25** and π-allyl complex **28** were heated to 50 °C for 16 h to form alkylated product **29** in 52% yield (Scheme 13A). MK-9 was produced in 85% yield and 7:3 *E/Z* using the same methods of deprotection and oxidation (Scheme 13B).

**Scheme 14:**
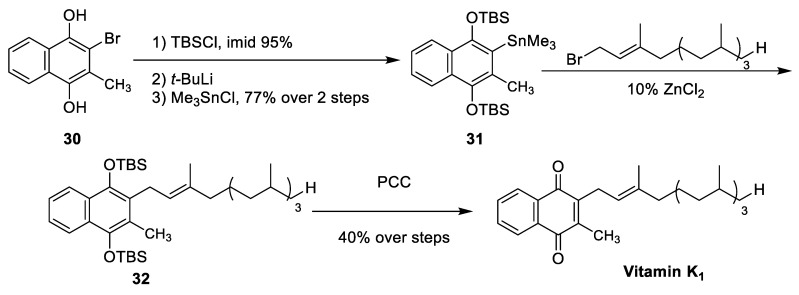
Synthesis of vitamin K_1_ using Stille organostannane chemistry [70].

Stille et al. published the synthesis of vitamin K_1_ using trimethylstannane derivatives to cross couple phytyl bromide in 1983 [70]. 2-Bromomenadiol **30** was protected with TBSCl in 95% yield (Scheme 14). Lithiation with *t*-BuLi and transmetalation with trimethyltin chloride formed **31** in 77% yield over two steps. Phytyl bromide was then coupled with **31** in the presence of ZnCl_2_ forming **32**. PCC was used to deprotect and oxidize the ring to form vitamin K_1_ in 40% yield over two steps.

**Scheme 15:**
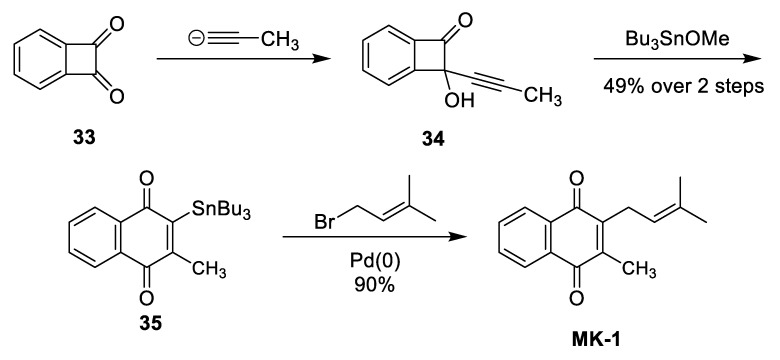
Synthesis of MK-1 featuring Liebeskind-Moore rearrangement to ring expansion [71].

In 1990, Liebeskind and Foster discovered an unexpected transformation that appeared useful towards the synthesis of MK-derivatives [71], which was previously mentioned in a review of syntheses of vitamin K and analogs [42]. MK-1 was synthesized using ring-strained dione **33** and propyne, forming alcohol **34** (Scheme 15). Then alcohol **34** underwent a Liebeskind-Moore rearrangement in the presence of Bu_3_SnOMe to form the stannylated product **35** in 49% over two steps. From there, MK-1 was synthesized in 90% yield using Stille cross-coupling conditions with Pd(0) and prenyl bromide.

### 3.2. Coordination Complex

**Scheme 16:**
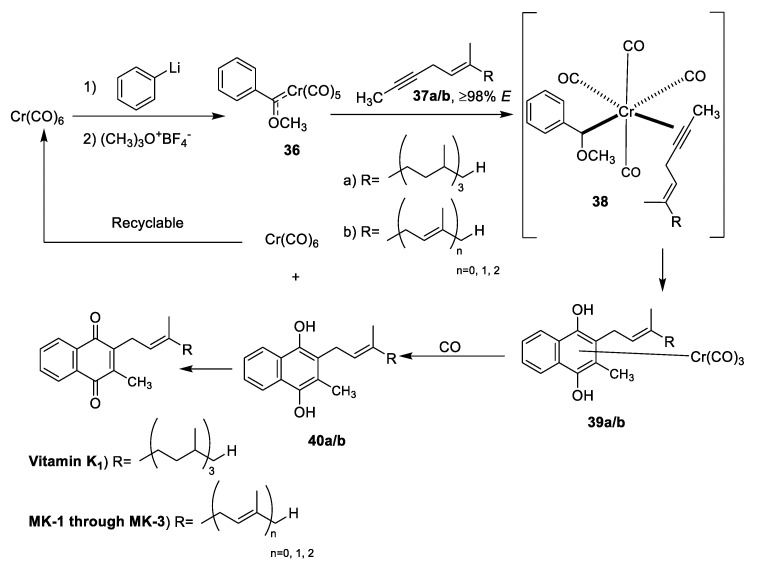
Synthesis of MK-derivatives using unique Cr(CO)_6_ mediated ring formation [43,72,73].

An intriguing approach to the synthesis of MK-derivatives was developed by Dötz et al. in 1986. Using pentacarbonyl(methoxyphenylcarbene)chromium(0) complex **36** and an alkyne (Scheme 16), the quinone ring was formed via carbonylation [43,72,73]. This approach provided access to several different naphthoquinone derivatives using functionalized alkynes. The authors synthesized vitamin K_1_ and MK-1 through MK-3 to illustrate this transformation. Phenyllithium reacted with one of the carbonyl ligands (CO) on Cr(CO)_6_, and then was methylated by trimethyloxonium tetrafluoroborate to form complex **36**. Vitamin K_1_ and MK-1 through MK-3 were formed by using alkynes **37a** or **37b**, respectively. Upon addition, the alkyne displaced another molecule of CO, to form intermediate complex **38**. The resulting menadiol ring **39a/b** was coordinated to Cr(CO)_3_. To make this route more sustainable, the authors determined Cr(CO)_6_ could be regenerated by pressurizing the system with CO to displace Cr(CO)_3_ and liberate the alkylated product **40a/b**. Oxidation with standard oxidizing agents afforded the menaquinone products accordingly. The yields of each reaction were not reported in Rüttimann’s 1986 review [43].

**Scheme 17:**
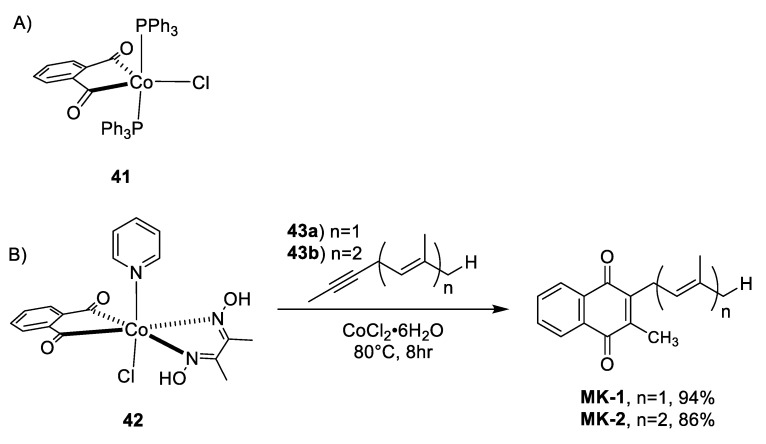
(**A**) Original cobalt complex synthesized by Liebeskind et al. for this transformation. (**B**) Synthesis of MK-1 and MK-2 using the updated catalyst [74,75].

In 1980, Liebeskind et al. designed a synthesis of naphthoquinones using bis(triphenylphosphine)phthaloylcobalt complex **41** (Scheme 17A). Ring formation was achieved upon addition of an alkyne in the presence of AgBF_4_ [74]. In 1986, an update was published wherein the authors describe an improved cobalt complex to increase the yield of the reaction and minimize the amount of AgBF_4_ required [75]. The updated complex **42** replaced the triphenylphosphine ligands with pyridine and dimethylglyoxime (Scheme 17B).

This new complex was more tolerant towards different Lewis acids as well as hydrated salts, which was demonstrated by the formation of **44** using complex **42**, as shown in Table 5. MK-1 and MK-2 were synthesized using alkynes **43a** and **43b**, cobalt complex **42** and Lewis acid in the form of CoCl_2_∙6H_2_O at 80 °C producing MK-1 and MK-2 in 94% and 86% yield, respectively.

### 3.3. Radical Reactions

#### 3.3.1. Metal-Mediated Radical Reactions

**Scheme 18:**
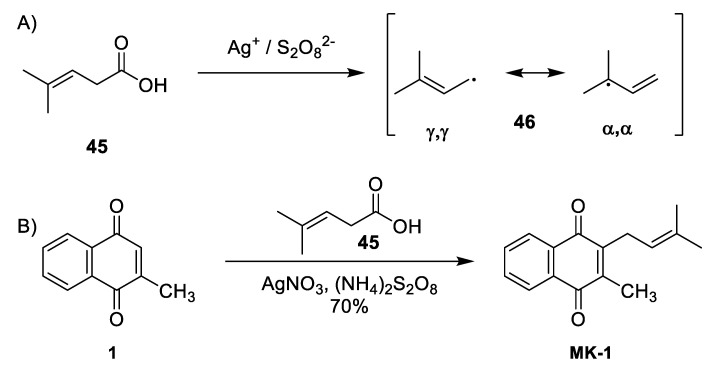
(**A**) Radical formation via decarboxylation. (**B**) Synthesis of MK-1 using this method [76,77].

In 1972, Jacobsen and Torssell reported the use of allyl radicals produced via decarboxylation of carboxylic acids to alkylate quinones. Upon mixing silver nitrate and ammonium peroxodisulfite, Ag^+^ and S_2_O_8_^2^ produce the radical, Ag^2+^ as shown in Equation (1). Then Ag^2+^ abstracts an electron from the carboxylic acid to produce CO_2_ and a radical species **R** as shown in Equation (2) [76]. The authors were concerned about possible rearrangement of the position of isoprene double bond [77]. 4-Methyl-3-pentenoic acid **45** formed the 3,3-dimethylallyl radical **46**, which resonates between α,α and γ,γ positions (Scheme 18A).

Ag^+^ + S_2_O_8_^2−^ → Ag^2+^ + SO_4_^∙−^ + SO_4_^2−^ (1)

Ag^2+^ + RCOOH → R^∙^ + CO_2_ + H^+^ + Ag^+^ (2)

**Scheme 19:**
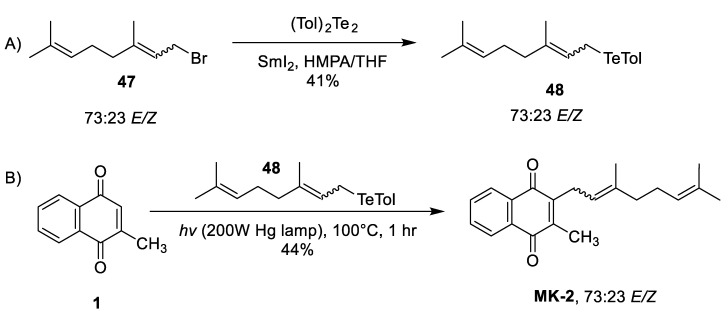
(**A**) Synthesis of geranyltelluride reagents. (**B**) Synthesis of MK-2 with organotelluride radical alkylation [78,79].

The more stable tertiary radical, α,α-dimethylallylquinone, was expected; however, only γ,γ-dimethylallylquinone was observed, favoring the more stable alkene. MK-1 was produced in 70% yield using **45** (Scheme 18B), leaving the question of *E/Z* alkene isomerization unanswered:

The question of α-isoprene double bond stereoretention was later answered by Yamago et al. in 2000 with preliminary results of the radical coupling of quinones with organotellurium reagents [78,79]. Geranyl bromide (73:23 *E/Z*) **47** was converted to the corresponding tolyltelluride **48** in 41% yield with complete retention of stereochemistry (Scheme 19A). Then the tolyltelluride **48** was photochemically coupled to menadione **1** to produce MK-2 in 44% yield with complete retention of stereochemistry (Scheme 19B). This reaction was previously mentioned in a review by Daines et al. in 2003 [42].

#### 3.3.2. Non-Metal-Mediated Radical Reactions

**Scheme 20:**
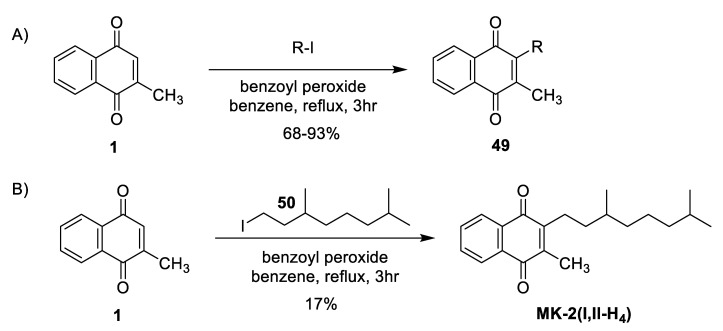
(**A**) General synthesis of menaquinones with alkyl iodides [80]. (**B**) Synthesis of MK-2 (I,II-H_4_) [38].

In 2019, we continued our pursuit to synthesize menaquinone analogs with various levels of saturation within the side chain [38]. For analogs with the first isoprene unit saturated, we employed chemistry developed by Coppa et al. in 1991. Therein, different methods for homolytic methylation of quinones with alkyl iodides were discussed [80]. In one such method, menadione **1** reacted with a saturated alkyl iodide in the presence of benzoyl peroxide to form the C3-alkylated product **49** (Scheme 20A).

The results varied, with yields ranging from 68 to 93%. Prominent competition between the C3-alkylated product and the C3-self-coupling aryl product was observed, showing 32–94% the C3 aryl product in some trials. We saw the parallels between the substrates used by Coppa et al. and the saturated prenyl side chains required for our studies. Menadione **1** was coupled with alkyl iodide **50** to synthesize MK-2(I,II-H_4_) in 17% yield (Scheme 20B).

### 3.4. Summary

The three main metal-mediated and radical methods reported in the literature included organometallic cross-coupling, the use of coordination complexes, and both metal and non-metal mediated radical reactions. The advantages and disadvantages of each have been outlined in Table 6. Only four methods reported moderate to high yields (65–100%) across the synthesis. Liebeskind and Foster used Stille coupling for the key alkylation step (90%). Liebeskind et al., again, reported high yields (77%) using the updated cobalt complex to aid the cycloaddition of alkynes to the coordinated phthaloyl group. Jacobsen and Torssell achieved moderate yields (70%) in the alkylation of quinones with radicals generated by the decarboxylation of prenyl carboxylic acids. Lastly, Coppa et al. also used radical alkylation of quinones using short-chain alkyl iodides in the presence of benzoyl peroxide. Regiocontrol for these methods was achieved in three ways: (1) transmetalation of organolithium reagents to different organometallates; (2) asymmetric alkynes coordinating to symmetric complexes; and 3) selective abstraction of aryl hydrogens adjacent to the carbonyl. Complete stereoretention was observed during Stille et al.’s cross-coupling of arylstannanes and phytyl bromide in the presence of ZnCl_2_, Dötz et al.’s cycloaddition using stereopure alkynes, and 

## 4. Electrophilic Ring Methods

### 4.1. 1,2-Addition versus 1,4-Addition

**Scheme 21:**
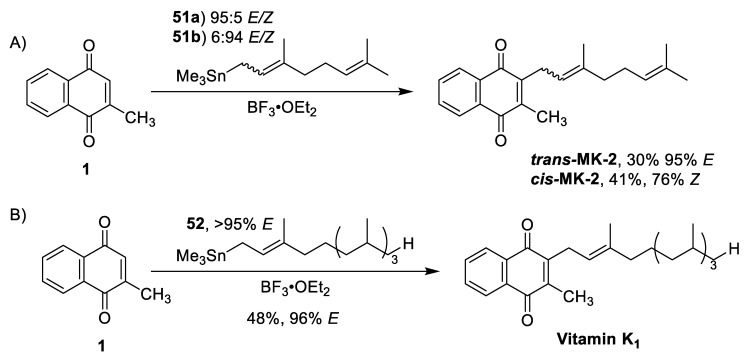
The synthesis of (**A**) MK-2 and (**B**) vitamin K_1_ using alkylstannanes [81,82,83].

In the late 1970s and early 1980s, Naruta published two reports independently and published one with Maruyuma, reporting the use of prenyl stannanes for the synthesis of biologically important quinones, focusing on vitamin K_1_ and MK-2 [81,82,83]. Using menadione **1**, *trans* and *cis* isomers with respect to the α-isoprene double bond were synthesized using geranyl and neryl trimethylstannanes, **51a** and **51b**, respectively, in the presence of BF_3_∙OEt_2_ (Scheme 21A). The yields were 30% and 41% for the respective *trans* and *cis* products with the configuration of the double bond mostly maintained for each product. *Trans*-MK-2 was found to have 95% *E* alkene, and the *cis* isomer was found to have 76% *Z* alkene. Using the same conditions, vitamin K_1_ was synthesized in 48% using phytyl trimethylstannane **52** (> 95% *E*) (Scheme 21B). The product was found to have 96% *E* alkene configuration, showing complete stereoretention.

Although the major product for all trials was the 1,4-addition product, 1,2-addition accounted for a large portion of the undesired byproducts. For *cis*-MK-2 and vitamin K_1_, the C2 isomer was isolated in 13% and 14% yield, respectively. The 1,2 addition is hypothesized to add the least hindered carbonyl carbon of menadione **1**, which then undergoes a [3,3] sigmatropic rearrangement, similarly described by Araki et al. [84] and Evans and Hoffmann [85] in Section 5.3. The rearrangement places the prenyl chain on the C2 carbon with the preexisting methyl group. In addition to prominent mechanistic competition, the yields are low across all steps. This method does, however, feature complete retention of stereochemistry of the α-isoprene double bond, as detailed in Table 7.

## 5. Pericyclic Reactions

### 5.1. Diels-Alder Reactions

**Scheme 22:**
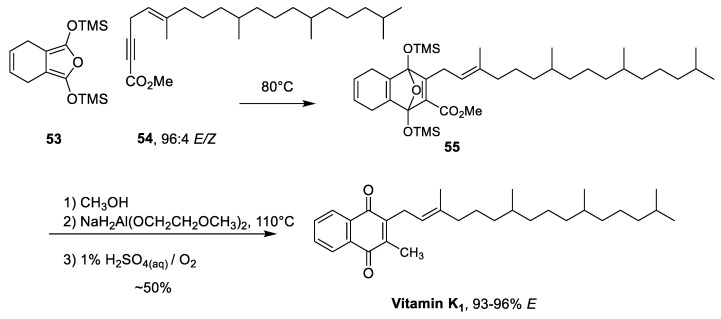
Previously unpublished synthesis of vitamin K_1_ inspired by Troll & Schmid [43].

In a review published by Rüttimann in 1986 [43], synthetic advancements of the preparation of vitamin K_1_ were presented, detailing methods from traditional substitutions, organometallic reactions, and pericyclic reactions. Therein, Rüttimann and coworkers explored the use of Diels-Alder reactions to form the naphthoquinone unit of vitamin K in a previously unpublished synthesis inspired by the work of Troll and Schmid. [86]. Dihydroisobenzofurane **53** was reacted with activated alkyne dienophile **54** (96:4 *E/Z*) at 80 °C overnight to form the Diels-Alder adduct **55** (Scheme 22). Deprotection of the silyl ethers was achieved using methanol, and reduction of C2 methyl ester to a methyl group with sodium bis(2-methoxyethoxy)aluminum hydride in toluene under reflux formed the substituted menadiol. Oxidation with air in slightly acidic conditions produced vitamin K_1_ in ~50% yield over four steps. The configuration of the isoprene double bond was not disturbed, resulting in 93–96% *E* alkene.

**Scheme 23:**
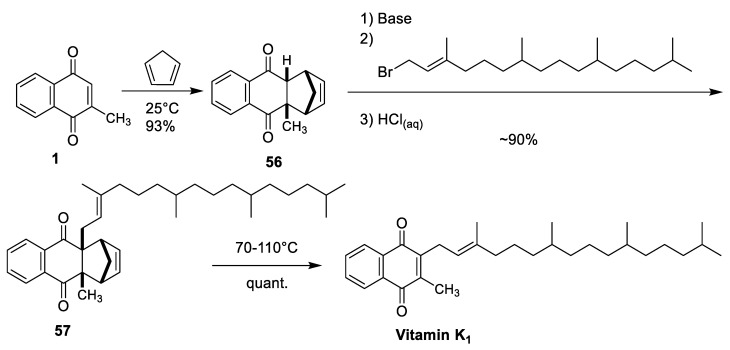
Synthesis of vitamin K_1_ using Diels-Alder approach with cyclopentadiene auxiliary [43].

Using insights gained from the previous route, Rüttimann continued to explore the use of Diels-Alder reactions to synthesize vitamin K_1_. He and Büchi designed an auxiliary-directed route using cyclopentadiene as the corresponding diene (Scheme 23) [87]. *Endo*-Diels-Alder adduct **56** was formed at room temperature using menadione **1** and cyclopentadiene in 93% yield. Formation of adduct **56** switched the C3 hybridization from sp^2^ to sp^3^_,_ decreasing the pKa. Upon deprotonation by a strong base (e.g., potassium amide, sodium amide, or potassium *t*-butoxide (which is referred to as potassium *t*-butanolate in the source literature)) a stable carbanion is formed, allowing regioselective alkylation at the C3 position with a variety of electrophiles.

Alkylation with phytyl bromide (≥98% *E*) gave the predicted product **57**. The authors found that some *O*-alkylation product was formed in trace amounts, however it was cleaved with acidic aqueous work up and easily separated. Alkylated adduct **57** was formed in approximately 90% yield over three steps. Due to the intrinsic instability of adduct **57**, slow decomposition was observed at room temperature. Retro-Diels-Alder reaction was induced at high temperatures to remove the auxiliary group quickly, producing vitamin K_1_ in quantitative yield [87].

### 5.2. Anionic Diels-Alder Reactions

**Scheme 24:**
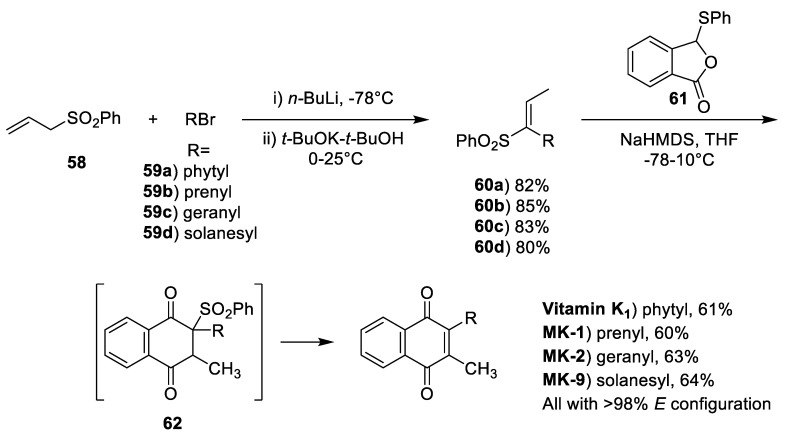
Synthesis of vitamin K_1_, MK-1, 2, and 9 using anionic Diels-Alder approach [88].

In contrast to traditional Diels-Alder reactions where neutral species form adducts, anionic Diels-Alder reactions have been another useful method to form the naphthoquinone unit. In 1995, Tso and Chen. published a one-pot synthesis applicable towards the synthesis of vitamin K_1_, and MKs-1, 2, and 9 (Scheme 24) [88]. The dienophiles used were alkenyl sulfones **60a–d**, which were readily prepared from the corresponding allyl phenylsulfone **58** and allyl bromides **59a–d** for vitamin K_1_, MK-1, MK-2, and MK-9, respectively. Isobenzofuranone **61** was deprotonated with NaHMDS, then **60a-d** were attacked forming the intermediate **62**. Elimination of the benzenesulfonate produced the desired products vitamin K_1_, MK-1, MK-2, and MK-9 in moderate yields, 60–64%. The configuration of the α-isoprene unit was found to be >98% *E* alkene for all substrates.

**Scheme 25:**
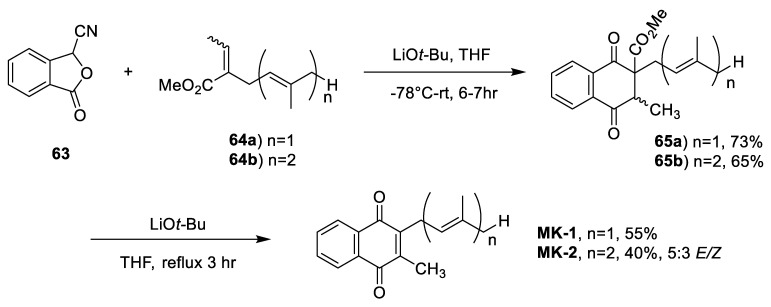
Synthesis of MK-1 and MK-2 using anionic Diels-Alder approach with improved atom economy [89].

Nearly twenty years later, Mal et al. suggested better atom economy could be obtained in comparison to the work done by Tso and Chen. In an effort to improve the atom economy of anionic Diels-Alder reactions, the authors replaced the phenylsulfone moiety with a nitrile **63** (Scheme 25) [89]. Using this method, the authors synthesized different C3-alkylated MK-derivatives using specifically designed dienophiles. For the synthesis of MK-1 and MK-2, methyl acrylate derivatives **64a** and **64b** were used. It is important to note the synthesis of **64b** produced a mixture of ~3:1 of *E/Z* isomers with respect to the methyl acrylate alkene, as determined by NMR. It is unclear to us whether that refers to stereochemical composition of the methyl acrylate alkene or the geranyl side chain of **64b**. Isobenzofuranone **63** was deprotonated with LiO*t*-Bu at −78 °C in THF, and then formed the adduct **65a/b** with dienophiles **64a/b** in 73% and 65% yield, respectively. Demethylcarboxylation of adducts **65a/b** was achieved using a second round of LiO*t*-Bu in THF under reflux to form MK-1 and MK-2 in 55% and 40% yield, respectively. The ratio of *E/Z* ratio of MK-2 was found to be 5:3.

### 5.3. [3,3] Sigmatropic Rearrangements-Cope

**Scheme 26:**
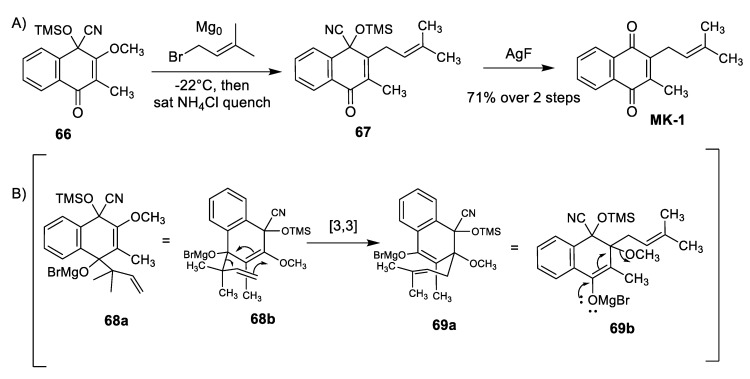
(**A**) Synthesis of MK-1 using prenylmagnesium bromide to Cope rearrangement. (**B**) Specific mechanism of the Cope rearrangement [85].

[3,3] Sigmatropic rearrangements were also used to synthesize menaquinones, as shown in Daines et al. 2003 review [42]. In 1976, Evans and Hoffmann took advantage of the Cope rearrangement to synthesize MK-1 (Scheme 26A) [85]. Using masked ketone **66**, the unprotected carbonyl was attacked with prenylmagnesium bromide, forming ketone **67** in one step. Interestingly, the authors discovered that prenylmagnesium bromide added to the carbonyl in a reverse prenylated fashion **68a** (Scheme 26B). The C1-reverse prenylated product **68a** set the transition state **68b** for a Cope rearrangement, **68b** to **69a**, producing C3-prenylated product **69a**. Upon quenching with saturated NH_4_Cl, as shown in intermediate **69b**, the C3 methoxy group was removed to reform ketone **67** (Scheme 26B). Deprotection of the masked ketone was achieved using AgF in mild conditions to form MK-1 in 71% yield over two steps. Since MK-1 was the only product synthesized, there *E/Z* ratio of the α-isoprene double bond was not considered, nor were the effects of this synthesis on longer prenyl chains.

**Scheme 27:**
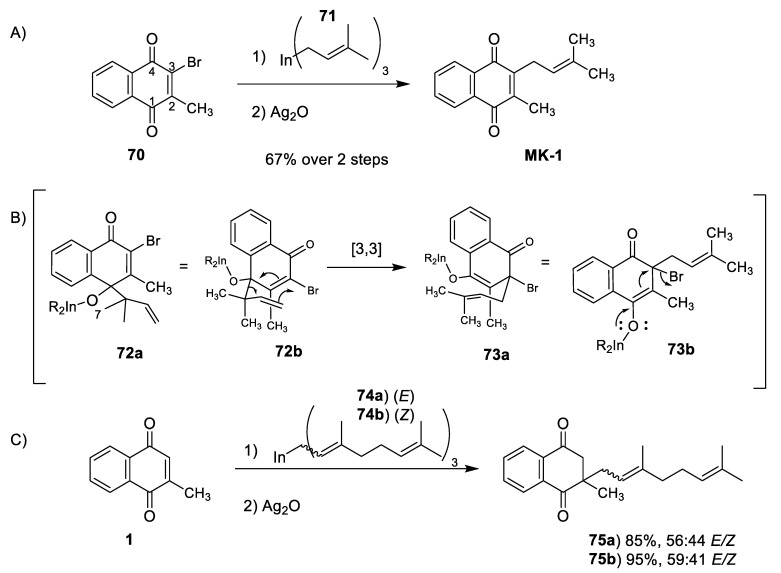
(**A**) Synthesis of MK-1 using organoindium reagents. (**B**) Closer look into the Cope rearrangement. (**C**) Attempted synthesis of MK-2 using geranyl **74a** and neryl **74b** organoindium rearrangement. [84].

In contrast to more conventional alkylating reagents, Araki et al. used organoindium reagents for the allylation of various quinones [84]. Throughout many trials with a wide scope of benzo- and naphthoquinone derivatives, organoindium reagents were found to selectively add to the least hindered carbonyl in 1,2-addition (Scheme 27A). Therefore, when choosing between the C1 and C4 carbonyl groups, 2-bromomenadione **70** was attacked at the less hindered C1 ketone with prenylindium **71** to form reverse prenylated intermediate **72a** (Scheme 27B). Following addition, the reverse prenyl group underwent a Cope rearrangement, **72b** to **73a**, analogous to the system reported by Evans and Hoffmann. After oxidation with Ag_2_O, MK-1 was produced in 67% over two steps. This synthesis did not address the possibility of alkene isomerization, however, in other trials reported in the same paper [84], the authors reported geranylindium **74a** and nerylindium **74b** rearrangements on menadione **1**, producing **75a** and **75b** in 85% and 95% yield, respectively (Scheme 27C). Each product showed a total loss of stereoretention, ~50/50 *E/Z*. The authors hypothesized the same trend would have been seen using 2-bromomenadione **70**; therefore, stereocontrol of the α-isoprene double bond continues to be an important challenge with Cope rearrangements.

### 5.4. Summary

Across the literature, the common types of pericyclic reactions are Diels-Alder cycloadditions and [3,3] sigmatropic rearrangements, specifically Cope rearrangements. The advantages and disadvantages of each method have been outlined in Table 8. Rüttimann et al. achieved high yields with a cyclopentadiene auxiliary-directed Diels-Alder reaction. For all methods described in this section, the asymmetry of certain reagents controlled the regioselectivity of the reaction. For example, the asymmetry of the dienophiles involved in the Diels-Alder reactions controlled the regiochemistry of the adduct. The Cope rearrangement reactions were regiocontrolled by using starting materials with leaving groups on the desired position, protecting groups, and by taking advantage of steric hinderance. For all Diels-Alder reactions described, except for the work done by Mal et al., complete retention of stereochemistry was observed for the α-isoprene double bond. In the case of Mal et al., the reported *E/Z* ratio for dienophile **64b** was ~3:1 *E/Z* by NMR for the methyl acrylate dienophile. The *E/Z* ratio of the product MK-2 was found to be 5:3 *E/Z.* It is unclear to us whether this was a result of the Diels-Alder reaction or due to isomeric starting material. For all Cope rearrangements, it can be inferred that a complete loss of stereocontrol would be observed due to the reverse-prenyl addition to the carbonyl, as observed by Araki et al. using geranyl and nerylindium reagents with menadione **1**.

## 6. Homologation & Side Chain Extension Methods

### 6.1. Homologation

**Scheme 28:**
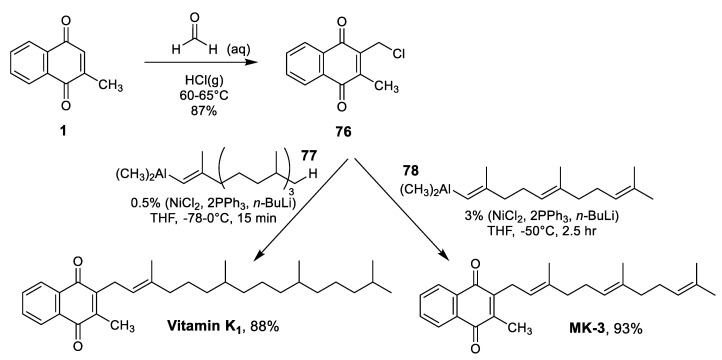
Synthesis of MK-3 and vitamin K_1_ via C3 homologation and Negishi cross-coupling conditions [90].

**Scheme 29:**
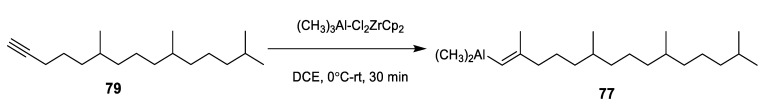
Negishi carboalumination of phytyl alkyne [91,92,93,94].

In 1998, Lipshutz et al. designed a route to install a one carbon functional handle at the C3 position to enable the synthesis of a wide variety of MK-derivatives [90]. Menadione **1** was reacted with formaldehyde and hydrogen chloride gas to form 3-chloromethylmenadione **76** in 87% yield (Scheme 28). The introduction of the chloromethyl group allows for reactions that were previously less approachable, like S_N_2 substitutions and organometallic cross-couplings. Using 3-chloromethylmenadione **76** as the starting material, the authors used Negishi cross-coupling conditions to take advantage of the stereoselective installation of alkenes based on the configuration of the organoalane species [91,92,93,94]. For example, phytyl alkyne **79** underwent Negishi carboalumination to form organoalane **77** (Scheme 29). Vitamin K_1_ and MK-3 were synthesized using this method (Scheme 28). For the synthesis of vitamin K_1_, 3-chloromethylmenadione **76** was coupled with phytylalane **77** in the presence of the nickel catalyst which is formed in situ using nickel (II) chloride, triphenylphosphine, and *n*-BuLi. Vitamin K_1_ was formed in 88% with exclusively *E* configuration. MK-3 was prepared similarly, but instead with farnesylalane **78**, in 93% yield with *E* configuration at the α-isoprene double bond.

**Scheme 30:**
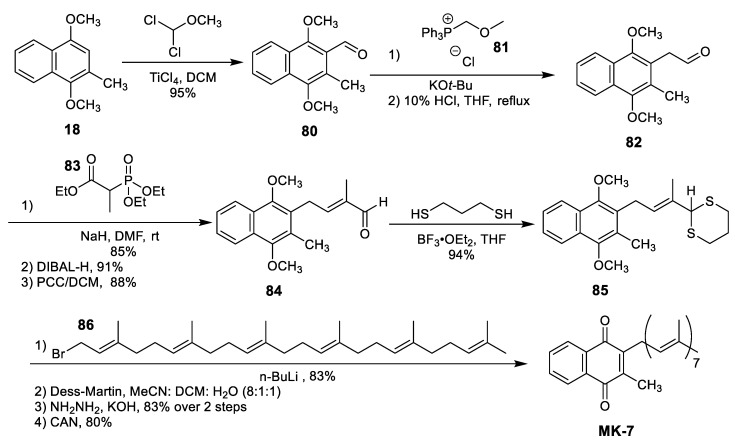
Synthesis of MK-7 using dithiane anion side chain extensions [95].

In 2015, Mehta et al. published a patent covering the synthesis of stereospecific quinone derivatives [95]. Therein, the authors described methods used for the synthesis of the various lengths of prenyl side chains using a series of homologation and side chain extension reactions featuring stereoselective alkene syntheses, such as Wittig, Horner-Wadsworth-Emmons, and Still-Gennari. For the synthesis of MK-7, 1,4-dimethoxynaphthoquinol **18** reacted with dichloro(methoxy)methane and TiCl_4_ to form the C3 aldehyde **80** in 95% yield (Scheme 30). Wittig homologation of **80** was achieved using ylide **81** followed by mild acid hydrolysis to form aldehyde **82**. Using phosphonate ester **83**, aldehyde **82** underwent a Horner-Wadsworth-Emmons reaction to form the desired *E* alkene in 85% yield. The authors noted the use of Still-Gennari conditions for the synthesis of the *Z* alkene where appropriate. The ester was reduced to the alcohol using DIBAL-H in 91% yield, and then immediately oxidized to aldehyde **84** using PCC in 88% yield. The resulting aldehyde **84** was protected as dithiane **85** in 94% yield. Deprotonation of the methine hydrogen of **85** with *n*-BuLi created a stable anionic nucleophile. Farnesylfarnesyl bromide **86** was used to form the C3-alkylated product in 83%. The dithiane protected carbonyl was deprotected using Dess-Martin periodinane and then reduced using Wolff-Kishner conditions, producing the hydrocarbon prenyl side chain in 82% yield over two steps. Oxidation with CAN formed MK-7 in 80% yield. The authors achieved the synthesis of MK-7 in two different iterations: (1) when the entire chain was added in one step (Scheme 30), and (2) when the side chain was added on smaller segments using the same methodology, which is similarly used in Masaki et al.’s work in Section 6.2.

### 6.2. Side Chain Extensions

**Scheme 31:**
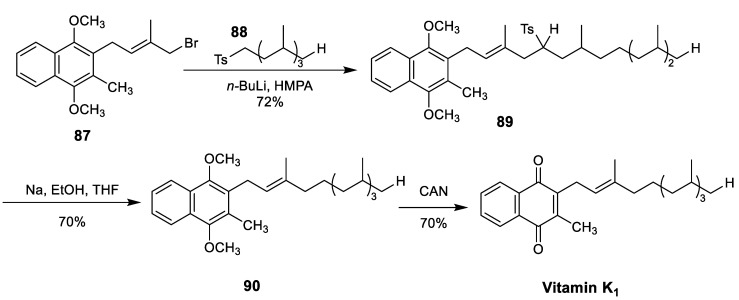
Synthesis of vitamin K_1_ using tosylate extension methods [96,97].

**Scheme 32:**
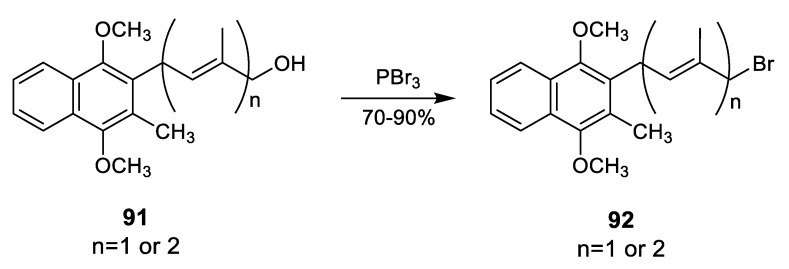
Conversion of prenyl alcohols to respective prenyl bromides [96,97].

**Scheme 33:**
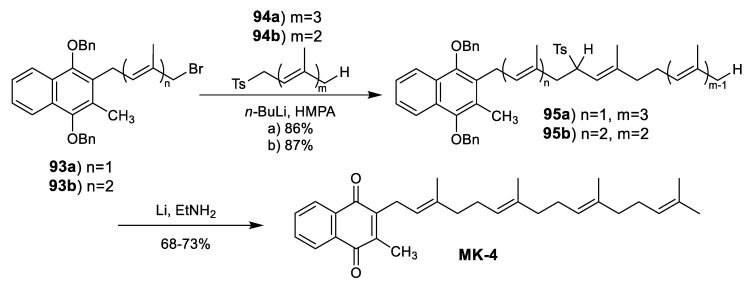
Synthesis of MK-4 using tosylate extension methods [96,97].

Another popular method across the literature is the continued functionalization of truncated prenyl chains that were installed using the previously described methods. In 1984, Masaki et al. developed a synthetic route to lengthen the prenyl chain starting with prenyl bromides (Scheme 31) [96,97]. This served as the starting material for the synthesis of vitamin K_1_ and MK-4. The common electrophiles across all trials were prenyl bromides **92**, synthesized in 70–90% yield from the corresponding prenyl alcohols **91** using PBr_3_ (Scheme 32). For the synthesis of vitamin K_1_ (Scheme 31), prenyl bromide **87** was coupled to tosylate **88**. Deprotonation of the tosylate methine hydrogen created a stable anion to attack **87** to form the alkylated product **89** in 72% yield. Removal of the tosyl group was achieved using modified Bauvault-Blanc desulfurization conditions in 70% yield. Deprotection of tosylate **89** obtained **90** in 70% yield, and thus vitamin K_1_ was produced in 70% yield in the presence of CAN. For the synthesis of MK-4, Masaki et al. approached the synthesis with two different iterations (Scheme 33). Starting with benzyl ether protected polyprenyl bromide **93a** and **93b**, the same alkylating conditions were used to extend the chain using tosylates **94a** and **94b** to form products **95a** and **95b** in 86% and 87% yield, respectively. Desulfurization of the prenyl chain produced the hydrocarbon side chain of MK-4 in 68–73% yield. The authors noted HPLC analysis showed isomeric byproducts in 5–7%, likely formed during the desulfurization step.

**Scheme 34:**
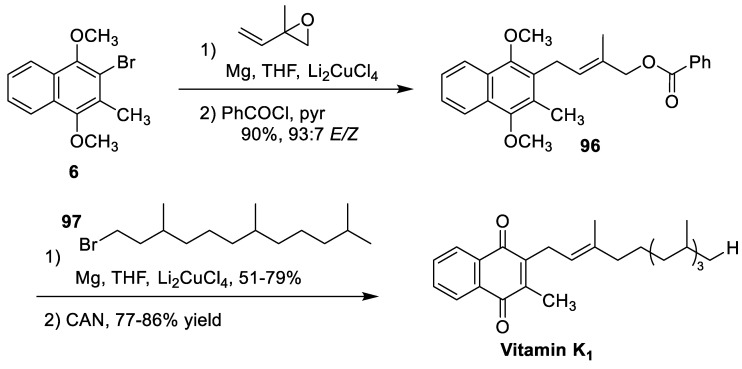
Synthesis of vitamin K_1_ using organocuprate reagents to extend the length of the side chain [63].

In contrast to Masaki et al.’s. approach with tosylates, Schmid et al. used organocuprate reagents as nucleophiles to install the remaining hydrocarbon chain for vitamin K_1_ [63]. Installation of the first prenyl group was achieved via coupling of organocuprate reagent of bismethyl ether 2-bromomenadiol **6** with isoprene oxide in a 1,4-addition (Scheme 34). The resulting alcohol was protected as ester **96**. This reaction yielded 90% over two steps with 93:7 *E/Z* configuration. The rest of the phytyl chain was installed using the organocuprate reagent formed with hexahydrofarnesyl bromide **97**, performing a S_N_2 substitution with the ester protecting group, forming the extended side chain in 51–79% yield. Oxidation of the ring was achieved with CAN with 77–86% yield to form vitamin K_1_.

### 6.3. Summary

Homologation and side chain extension methods comprise a separate category from the others despite the use of similar techniques because they provide a functional handle that enables more diverse reactions that were previously less accessible. Homologation describes the addition of one carbon-containing group to the C3 position, which is then further functionalized to synthesize the rest of side chain. Side chain extension methods have one or two isoprene units attached to the ring which were installed using previously described methods to then add the remainder of the chain using a different method, allowing for continued iterative additions. The advantages and disadvantages of each method have been outlined in Table 9. Nearly all described methods produced moderate to high yields throughout all steps, except for the organocuprate substitution demonstrated by Schmid et al., which produced low yields compared to the rest of the synthesis. Regiocontrol for these methods was achieved by the preinstalled carbons or prenyl chains connected to the ring which make the subsequent reactions chemoselective. Stereoretention of the α-isoprene double bond was achieved in three ways: (1) using stereospecific methodology, like Negishi carboalumination and cross-coupling; (2) stereoselective alkene syntheses; and (3) taking advantage of the stereochemical outcome of S_N_2 substitutions.

## 7. Conclusions

Menaquinones are a biologically important class of isoprenoid compounds that comprise of a methylated naphthoquinone unit and an isoprene side chain of various lengths and levels of saturation. The intrinsic hydrophobicity of these compounds makes it exceedingly difficult to analyze the activity in aqueous-based in vitro assays and growth studies. The moderately water soluble, truncated derivatives, MK-1 through MK-3, have made it possible to test and measure the activities of these compounds, especially in membrane-associated proteins. The most popular method used to synthesize menaquinones and derivatives has been BF_3_∙OEt_2_-catalyzed Friedel-Crafts reaction. This reaction typically occurs over two steps from commercially available starting material, but it generally produces low yields and a mixture of isomers. Although Friedel-Crafts alkylation is the conventional, concise method, in this review we aimed to compile and evaluate syntheses of menaquinones from all the literature to summarize the advantages and disadvantages of each route based on overall yield, regioselectivity, and stereoretention of the α-isoprene double bond, as shown in Table 4, Table 6, Table 7, Table 8 and Table 9.

We further evaluated the reactions to consider the number of steps in the synthesis, minimization of side reactions, and overall safety to highlight one representative method in each category, as outlined in Table 10. With examination of Table 10, it is possible to compare the syntheses across different approaches. As a representative of the nucleophilic ring methods, Swenton and coworkers were chosen for the clean use of electrolysis to protect the quinone as bismethyl ketals. Simple acid hydrolysis deprotection of the ketals reformed the carbonyls of the quinone without oxidizing agents. As a representative of the metal-mediated and radical reactions, Liebeskind et al. were chosen for the easily synthesized cobalt complex and internal alkynes. The updated cobalt complex was found to be more tolerant toward a wide scope of Lewis acid catalysts, and, as a result decreased the amount of catalyst required to synthesize the product. Rüttimann and Büchi designed an auxiliary-directed Diels-Alder reaction using commercially available materials, cyclopentadiene and menadione. Alkylation of the adduct occurred chemoselectively, and then facile retro-Diels-Alder formed the desired product in high yields and excellent regioselectivity. Lipshutz et al. used homologation and Negishi carboalumination and cross-coupling as side chain extension methods to attach the prenyl side chain. The stereochemistry of the α-isoprene double bond was easily installed using Negishi carboalumination of the corresponding terminal alkyne. Finally, the representative electrophilic ring strategy was done in one synthesis of menaquinones using Michael additions was presented. The method developed by Naruta and Maruyuma retained the stereochemistry of α-isoprene double bond but was not regioselective due to competing 1,2- and 1,4-addition mechanisms.

Although the methods described in Table 10 are short, high yielding, and selective, there are a few more methods that should be considered if a new synthetic target can accommodate a longer synthesis or the processes in Table 10 are incompatible for the particular target. The homologation method developed by Mehta et al. uses two one-carbon installations at the beginning to synthesize provide a functional handle to stereoselectively install the α-isoprene double bond, and thus can be a strong competitor to the other reactions. Even though many of the methods described have great success using prenyl halides, the reagents are expensive, particularly in comparison to synthesizing MK-derivatives from cheap prenyl alcohols. Saá & coworkers were successful in using commercially available prenyl aldehydes as electrophiles (no distillation necessary). Reduction of the resultant benzyl alcohol was achieved using Birch hydrogenolysis conditions to maintain the stereochemistry of the α-isoprene double bond. Despite the notorious use of Friedel-Crafts alkylations, Schmid et al. discovered a unique intramolecular Friedel-Crafts alkylation beginning with the *O*-alkylated product using BF_3_∙OEt_2_. This route adds at two steps, not including synthesis of the starting material and one protecting group, but the additional steps improved the overall yield, as well as the regio- and stereochemistry. Lastly, even though Jacobsen and Torssell only synthesized MK-1, their method would most likely tolerate polyprenylcarboxylic acids based on the stereoretentive results of radical coupling with organotellurides reported by Yamago et al.

With respect to Michael additions, the method developed by Naruta and Maruyuma was plagued by competing mechanisms (1,2- vs. 1,4-addition). It appears that alkylstannanes are not chemoselective enough to produce the Michael product for C-C bonds; however, many advancements have been made regarding Michael additions in general, suggesting more regioselective Michael additions may now be available for the synthesis of menaquinone and derivatives. Recently, Michael additions have been successfully used to synthesize C-N bonds using primary amines to form new analogues for biological testing. Recently, Salunke-Gawali and coworkers have used *n*-alkylamines and aminophenols to synthesize naphthoquinone derivatives which have been reported to assess their antiproliferative and antibacterial activities [98,99]. Salunke-Gawali and coworkers used single crystal x-ray crystallography to determine the structures of these compounds. The incorporation of C-N bonds introduces more hydrogen bonding sites for both intra- and intermolecular interactions. Similarly, Zacconi et al. synthesized naphthoquinone derivatives using benzylamine and 2-phenylethylamine to evaluate their isothermal solubility in supercritical carbon dioxide [100]. All these MK-derived compounds were solids, compared to the typical oil produced for the all-C-atoms MK-derivatives. It would be of great interest to synthesize truncated menaquinones with *N*-containing isoprenyl side chains and study their redox potentials, solubility, and potential solid-state structure compared to the compounds already reported. However, as demonstrated by this review, many attractive strategies are available that could be used to synthesize potential targets even if specific geometries are required.

## Figures and Tables

**Table 1:** Yields for different acid catalysts used in the synthesis of vitamin K_1_ [54].

Acid Catalyst	% Yield ^1^
KHSO_4_	55%
Oxalic Acid	N/A ^2^
Duolite C-60	8%
BF_3_∙OEt_2_	66.5%

^1^ Over two steps. ^2^ No yield reported.

**Table 2:** Friedel-Crafts alkylation of **18** with sulfonyl **19** to form **20** [65].

Lewis Acid ^1^	% of 20 (*E/Z*)
BF_3_∙OEt_2_	0 (-)
MgBr_2_	0 (-)
TiCl_4_	- ^2^(-)
FeCl_3_	55 (4:1)
Et_2_AlCl	56 (7:1)
SnCl_4_	56 (*E*)
ZnBr_2_	60 (7:1)
ZnCl_2_	67 (7:1)
AlCl_3_	72 (*E*)

^1.^ 1.2 equiv of Lewis acid was used. ^2.^ Decomposition of the starting materials was observed.

**Table 3:** The catalytic results in the synthesis of vitamin K_1_, K_1_-chromanol, and C2-alkylated product from menadiol [66].

Catalyst	% of Vitamin K_1_	% of K_1_ Chromanol	% of C2 Product
MgF_2_-40	21.2	5.9	58.8
MgF_2_-57	26.5	21.0	43.7
MgF_2_-71	15.6	20.8	52.9
MgF_2_-87 ^1^	0	0	0
AlF_2_-50	7.6	42	41.2

^1^ 0% conversion.

**Table 4:** Summary of nucleophilic ring methods.

Methods	Advantages	Disadvantages
**Section 2.1. Enolate Alkylations**		
Snyder and Rapoport Enolate Alkylation [44]	-Stereoretention of α-isoprene double bond (97% *E*-alkene) -3 step synthesis (not including starting material)	-Low yields (20–45%) -C2 alkylation competition via Friedel-Crafts alkylation -Unviable synthesis of starting material
Tabushi et al. β-cyclodextrin inclusion catalyst [45,46]	-Regiocontrol via sterically hindered nature of β-cyclodextrin -Menadione is the only byproduct -1 step synthesis	-Low yields (40% with inclusion catalyst) -Competition between C3 alkylation and C3 protonation -Only synthesized MK-1
**Section 2.2. Transmetalations**		
Snyder and Rapoport Grignard reaction [44]	-Regiocontrol through lithium-bromide exchange -Stereoretention of the α-isoprene double bond (≥95%) -Alkylation step is high yielding (>95%) -3 step synthesis (not including starting material)	-Need to prepare starting material 6
Saá and coworkers BIHY Reduction [47,48,49]	-Stereoretention of the α-isoprene double bond during BIHY reduction	-Moderate yields for nucleophilic addition (58–65%) and BIHY reduction (53–70%) -5 step synthesis (not including starting material)
Swenton and coworkers Electrolysis & Lithium Organocuprate [50,51]	-Unique use of electrolysis as a protection method -High yields for all reported steps (≥85%) -Regiocontrol through lithium bromide exchange -Stereoretention of α-isoprene double bond (<5% *Z*-alkene estimated) -Deprotection of bisketals to menaquinone ring structure via hydrolysis, no oxidation required	-Lithium organocuprate nucleophile only used one of two bisketal rings—poor atom economy -Difficult purification because of unreacted starting materials -5 step synthesis (not including starting material)
**Section 2.3. Friedel-Crafts Alkylation**		
Hirschmann et al. Friedel-Crafts Alkylation Lewis Acid Analysis [54]	-Favors C3 alkylation over C2 due to monoacetate **14** -Avoided formation of undesired byproducts (phytadiene and chromanol) -Monoacetate **14** was the only recoverable byproduct -2 step synthesis (not including starting material)	-Low to moderate yields (8–66.5%) depending on acid catalyst used (Table 1) -Stereoretention of the α-isoprene double bond was not discussed
Schmid et al. Intramolecular Friedel-Crafts [63]	-Features unique intramolecular Friedel-Crafts alkylation at C3 position -High yields (76–96.5%) throughout all steps -Stereoretention of α-isoprene double bond -3 step synthesis (not including starting material)	-Need to prepare starting material **22**
Min et al. Friedel-Crafts Alkylation Lewis Acid Analysis [65]	-Stereoretention of α-isoprene double bond with AlCl_3_ -Produced a functional handle for chain extension methods -1 step synthesis (not including starting material)	-Low to moderate yields (0–72%) depending on Lewis acid used
Coman et al. [66] and Koehn et al. [38] Heterogenous Lewis Acid Catalysts	-Predicted industrial benefit to replace BF_3_∙OEt_2_ -Performed without protecting groups, but could benefit from them -1 step synthesis (not including starting material)	-Universally low yields (0–26.5%) -Poor regiocontrol to prevent C2 alkylation -Difficult purification -Synthesis of partly hydroxylated metal fluorides requires the use of dangerous aqueous HF [67,68]

**Table 5:**
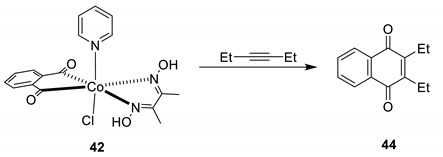
Effects of Additives on 2,3-diethyl-1,4-naphthoquinone **44** formation at 80 °C [75].

Additive (1 Equiv)	GC Yield % of 44		
	2 h	5 h	18 h
None	25	52	77
AgBF_4_	80	82	-
BF_3_∙OEt_2_	74	79	82
SnCl_2_	41	39	70
CoCl_2_∙6H_2_O	59	83	91
CoCl_2_(anhyd)	61	86	86
*p*-CH_3_PhSO_3_H	14	31	76
CH_3_CO_2_H	23	47	74

**Table 6:** Summary of metal-mediated reactions.

Methods	Advantages	Disadvantages
**Section 3.1. Cross-Coupling**		
Sato et al. π-Allylnickel Cross Coupling [69]	-No coordination complex synthesis required-π-allyl complex is formed in situ -Moderate to high yields (52–93%) across the syntheses -*E/Z* ratio of the α-isoprene double bond (7:3 *E/Z* for MK-9) -3 step synthesis (not including starting material)	-The yields drop at the cross-coupling, especially for the much longer prenyl side chains, MK-9 (52%) -Authors note *E/Z* ratio is tunable depending on the solvent, but the yields drop as a result
Stille et al. Aryl Stannane Cross Coupling [70]	-High yields (77%) for the formation of the arylstannane -The regiochemistry of the system is controlled by transmetalation at C3 position -Allylic transposition was not observed in analogous syntheses of myrcene [70]	-Low yield for cross-coupling (40% over two steps) -Requires the use of *t*-BuLi -5 step synthesis (not including starting material)
**Section 3.2. Coordination Complex**		
Liebeskind and Foster Ring Expansion to Stille Coupling [71]	-Stille coupling achieved high yields (90%) -3 step synthesis (not including starting material)	Low yield for key Liebeskind-Moore rearrangement (49% over two steps) -Only synthesized MK-1
Dötz et al. Chromium Complex Carbonylation [72,73]	-No coordination complex synthesis required -*E/Z* ratio of the α-isoprene double bonds was retained throughout the synthesis -The regiochemistry of the system is controlled by the alkynes **37a** and **37b** -Cr(CO)_6_ is recyclable	-Known adverse health effects related to hexavalent chromium -No yields reported in Rüttimann’s 1986 review [43] -5 step synthesis (not including starting material)
Liebeskind et al. Cobalt Complex Cycloaddition [75]	-High yields (>86%) -Simple coordination complex synthesis required using commercially available materials -The regiochemistry of the system is controlled by the alkynes **43a** and **43b** -1 step synthesis (not including catalyst)	-The authors did not address α-isoprene double bonds isomerization
**Section 3.3. Radical Reactions**		
Jacobsen & Torssell Radical Decarboxylation [77]	-Moderate yields (70%) -Regiocontrolled through aryl hydrogen abstraction -Selective for γ,γ-alkene product of MK-1 -1 step synthesis	-Only synthesized MK-1
Yamago et al. Radical Organotelluride [78,79]	-Regiocontrolled through aryl hydrogen abstraction -Stereoretention of the α-isoprene double bond across all steps -2 step synthesis	-Low yields for both formation of tolyltelluride and radical coupling (~40%) -Known adverse health effects related to working with tellurium and tellurium compounds
Coppa et al. [80] & Koehn et al. [38] Benzoyl Peroxide Initiated Radical Alkylation	-Moderate to high yields of straight chain alkyl iodides (68–93%) [80] -1 step synthesis (not including starting material)	-Koehn et al. reported very low yields (17%) for this transformation with a branched alkane -Substantial α-isoprene double bond isomerism -Competing reactions interfere with C3-alkylated product (C3-C3, and C2 alkylation)

**Table 7:** Summary of electrophilic ring methods.

Methods	Advantages	Disadvantages
Section 4.1. 1,2-Addition vs. 1,4-Addition		
Naruta and Maruyuma. Organostannane Michael Addition [81,82,83]	-Stereoretention of the α-isoprene double bond -1 step synthesis (not including starting materials)	-Low yields for both formations (30–48%) -Prominent competition between C2 and C3 alkylation

**Table 8:** Summary of pericyclic reactions.

Methods	Advantages	Disadvantages
**Section 5.1. Diels-Alder**		
Rüttimann et al. Diels-Alder Reaction inspired by Troll & Schmid [43]	-High regiocontrol through the symmetry of dihydroisobenzofurane diene -Stereoretention of α-isoprene double bond (≥ 93%)	-Overall low yields (~50% over four steps) -Synthesis of starting materials -4 step synthesis (not including starting material)
Rüttimann et al. Auxiliary-Directed Diels-Alder [43]	-Uses commercially available starting materials (menadione and cyclopentadiene) -High regiocontrol through adduct **66** -Stereoretention of α-isoprene double bond -Cyclopentadiene can be recycled -High yields throughout the synthesis (≥90%)	-Slight competition between C-alkylation and O-alkylation -5 step synthesis
**Section 5.2. Anionic Diels-Alder**		
Tso and Chen Anionic Diels-Alder [88]	-One-pot synthesis -High regiocontrol through asymmetry of dienophile -Stereoretention of α-isoprene double bond across all steps (>98%) -3 step synthesis (not including starting material)	-Moderate yields (60–64%) -Requires the synthesis of starting materials
Mal et al. Anionic Diels-Alder with Improved Atom Economy [89]	-Improved atom economy -High regiocontrol through asymmetry of dienophile -2 step synthesis (not including starting material)	-Low to moderate yields (40–73%) -5:3 E/Z ratio -Unclear if it is due to stereochemistry of starting material or caused by the reaction
**Section 5.3. [3,3] Sigmatropic Rearrangements- Cope**		
Evans and Hoffmann Grignard-Promoted Cope Rearrangement [85]	-Regiocontrol achieved through protected naphthoquinone -Cope rearrangement to achieve C3 alkylation -Moderate yields (71% over two steps) -2 step synthesis (not including starting material)	-No consideration of the isomerization of the isoprene double bond
Araki et al. Organoindium-Promoted Cope Rearrangement [84]	-Regiocontrol achieved through less hindered 1,2-addition of organoindium reagent -No protecting groups required -Cope rearrangement to achieve C3 alkylation -Moderate yields (67% over two steps) -2 step synthesis (not including starting material)	No stereoretention observed in Cope rearrangement

**Table 9:** Summary of homologation and side chain extension methods.

Methods	Advantages	Disadvantages
**Section 6.1. Homologation**		
Lipshutz et al. Homologation to Negishi Cross-Coupling [90]	-High yields throughout the synthesis (87–93%) -Method is applicable to a wide scope of benzo- and naphthoquinones -Stereochemistry of the α-isoprene double bond is defined by the configuration of the organoalane -Regiocontrolled by the installation of the chloromethyl group at the C3 position -No extraneous coordination complex synthesis required -3 step synthesis (including starting material)	-Requires the use of hydrogen chloride gas
Mehta et al. Stereoselective Alkene Syntheses [95]	-High yields throughout the synthesis for all reported steps (80–95%) -Strict use of stereoselective alkene syntheses -Methodology is applicable to full side chain extensions and smaller segments	-Requires the use of protecting groups and oxidation manipulations -11 step synthesis (not including starting material)
**Section 6.2. Side Chain Extension**		
Masaki et al. Tosylate Substitution [96,97]	-Moderate to high yields throughout the synthesis (68–90%) Stereoretention of the α-isoprene double bond with minor isomerization (5–7%) Methodology is applicable to full side chain extensions and smaller segments	-4 step synthesis (not including starting material)
Schmid et al. Organocuprate Substitution [63]	-Achieved regio- and stereocontrol using isoprene oxide in a 1,4-addition -Stereoretention of the α-isoprene double bond (97:3) -Iterative methodology	-Low to moderate yields (51–79%) for alkylation step -4 step synthesis (not including starting material)

**Table 10:** Summary of the best reactions within each strategy to be compared to each other.

Strategy-	Advantages	Disadvantages
**Nucleophilic Ring***2.2. Transmetalation* Swenton and coworkers Electrolysis and Lithium Organocuprate [50,51]	-Unique use of electrolysis as a protection method -High yields for all reported steps (≥85%) -Regiocontrol through lithium bromide exchange -Stereoretention of α-isoprene double bond (<5% *Z* alkene estimated) -Deprotection of bisketals to menaquinone ring structure via hydrolysis, no oxidation required	-Lithium organocuprate nucleophile only used one of two bisketal rings—poor atom economy -Difficult purification because of unreacted starting materials -5 step synthesis (not including starting material)
**Metal-Mediated** *3.2. Coordination Complex* Liebeskind et al. Cobalt Complex Cycloaddition [75]	-High yields (>86%) -Simple coordination complex synthesis required using commercially available materials -The regiochemistry of the system is controlled by the alkynes **43a** and **43b** -1 step synthesis (not including catalyst)	-The authors did not address α-isoprene double bonds isomerization
**Electrophilic Ring** *4.1. 1,2- vs. 1,4-Addition* Naruta and Maruyuma Organostannane Michael Addition [81,83]	-Stereoretention of the α-isoprene double bond	-Low yields for both formations (30–48%) -Prominent competition between C2 and C3 alkylation
**Pericyclic** *5.1. Diels-Adler* Rüttimann et al. Auxiliary-Directed Diels-Alder [43]	-Uses commercially available starting materials (menadione and cyclopentadiene) -High regiocontrol through adduct **66** -Stereoretention of α-isoprene double bond -Cyclopentadiene can be recycled -High yields throughout the synthesis (≥90%)	-Slight competition between C-alkylation and O-alkylation
**Homologation & Side Chain Extensions** *6.1. Homologation* Lipshutz et al. Homologation to Negishi Cross-Coupling [90]	-High yields throughout the synthesis (87–93%) -Method is applicable to a wide scope of benzo- and naphthoquinones -Stereochemistry of the α-isoprene double bond is defined by the configuration of the organoalane -Regiocontrolled by the installation of the chloromethyl group at the C3 position -No extraneous coordination complex synthesis required	-Requires the use of hydrogen chloride gas

## Abbreviations

β-CD: β-cyclodextrin. BIHY: Birch hydrogenolysis. CAN: ceric ammonium nitrate. DCM: dichloromethane, methylene chloride. DIBAL-H: diisobutylaluminum hydride. EtOH: ethanol. HMDS: hexamethyldisilazane. HMDSZ: outdated term, hexamethyldisilazane. HMPA: hexamethylphosphoramide. HPLC: high performance liquid chromatography. Hν: light. Imid: imidazole. LiOt-Bu: lithium tert-butoxide. MeCN: acetonitrile. MeOH: methanol. MK: menaquinone, methylnaphthoquinone. NaHMDS: sodium hexamethylsilazide, sodium hexamethyldisilazane. n-BuLi: *n*-butyl lithium. PCC: pyridinium chlorochromate. PPh_3_: triphenylphosphine. Pyr: pyridine. S_N_2: substitution nucleophilic bimolecular. TBSCl: tert-butyldimethylsilyl chloride. t-BuLi: *t*-butyl lithium. t-BuOH: *tert*-butyl alcohol. t-BuOK: potassium *tert*-butoxide. THF: tetrahydrofuran. TMSCl: trimethylsilyl chloride. Tol: tolyl. U: ubiquinone, benzoquinone.
